# Control of Polar/Antipolar Layered Organic Semiconductors by the Odd‐Even Effect of Alkyl Chain

**DOI:** 10.1002/advs.202308270

**Published:** 2024-01-25

**Authors:** Satoru Inoue, Toshiki Higashino, Kiyoshi Nikaido, Ryo Miyata, Satoshi Matsuoka, Mutsuo Tanaka, Seiji Tsuzuki, Sachio Horiuchi, Ryusuke Kondo, Ryoko Sagayama, Reiji Kumai, Daiki Sekine, Takayoshi Koyanagi, Masakazu Matsubara, Tatsuo Hasegawa

**Affiliations:** ^1^ Department of Applied Physics The University of Tokyo Hongo Bunkyo‐ku Tokyo 113‐8656 Japan; ^2^ Research Institute for Advanced Electronics and Photonics (RIAEP) National Institute of Advanced Industrial Science and Technology (AIST) Tsukuba Ibaraki 305‐8565 Japan; ^3^ Department of Life & Green Chemistry Saitama Institute of Technology Fukaya Saitama 369‐0293 Japan; ^4^ Department of Physics Okayama University Okayama 700‐8530 Japan; ^5^ Photon Factory Institute of Materials Structure Science High Energy Accelerator Research Organization (KEK) Tsukuba Ibaraki 305‐0801 Japan; ^6^ Department of Physics Tohoku University Sendai 980‐8578 Japan; ^7^ Center for Science and Innovation in Spintronics Tohoku University Sendai 980‐8577 Japan; ^8^ PRESTO Japan Science and Technology Agency (JST) Kawaguchi 332‐0012 Japan

**Keywords:** alkyl chains, layered crystallinity, organic semiconductors, odd‐even effects, polar crystals

## Abstract

Some rodlike organic molecules exhibit exceptionally high layered crystallinity when composed of a link between π‐conjugated backbone (head) and alkyl chain (tail). These molecules are aligned *side‐by‐side* unidirectionally to form self‐organized polar monomolecular layers, providing promising 2D materials and devices. However, their interlayer stacking arrangements have never been tunable, preventing the unidirectional arrangements of molecules in whole crystals. Here, it is demonstrated that polar/antipolar interlayer stacking can be systematically controlled by the alkyl carbon number *n*, when the molecules are designed to involve effectively weakened *head‐to‐head* affinity. They exhibit remarkable odd–even effect in the interlayer stacking: alternating *head‐to‐head* and *tail‐to‐tail* (antipolar) arrangement in odd‐*n* crystals, and uniform *head‐to‐tail* (polar) arrangement in even‐*n* crystals. The films show excellent field‐effect transistor characteristics presenting unique polar/antipolar dependence and considerably improved subthreshold swing in the polar films. Additionally, the polar films present enhanced second‐order nonlinear optical response along normal to the film plane. These findings are key for creating polarity‐controlled optoelectronic materials and devices.

## Graphical abstract

Control of polar/antipolar molecular arrangements is demonstrated by newly designed rod‐like organic semiconductor molecules that take advantage of the odd–even nature of alkyl chain length. The materials enable the fabrication of highly homogeneous polar/antipolar single‐crystal thin films through a straightforward solution process. The films exhibit strong second‐order nonlinear optical response and excellent field‐effect transistor characteristics, demonstrating a unique odd–even dependence.

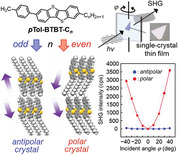

## 1 Introduction

The intermolecular arrangement is a crucial factor in determining the performance of optoelectronic devices based on π‐conjugated organic materials. Carrier transport characteristics as well as linear/nonlinear optical responses depend critically on the molecular packing motif as well as the completeness of macroscopic molecular order.^[^ ^1^, ^2^, ^3^ ^]^ However, control of the intermolecular arrangements – which is often called as crystal engineering – has been an important but quite difficult issue in materials science, since a subtle modification of molecular components, such as π‐conjugated backbones and their substituents, often causes drastic changes in the molecular packing motifs.^[^ ^4^, ^5^, ^6^, ^7^ ^]^ Highly deliberate design of the molecules is quite necessary for controlling the crystal structures of molecular materials toward ideal optoelectronic device applications.^[^ ^8^ ^]^

Among them, alkyl‐chain substitution can be the most effective way for obtaining and controlling 2D arrangements of π‐conjugated molecules.^[^ ^9^, ^10^, ^11^, ^12^, ^13^, ^14^, ^15^, ^16^, ^17^ ^]^ It was recently shown that some unsymmetric rodlike organic molecules, composed of a link between π‐conjugated backbone (π‐core) and alkyl chain, exhibit exceptionally high layered crystallinity. A typical molecule is phenyl‐/alkylated‐[1]benzothieno[3,2‐*b*][1]benzothiophene (Ph‐BTBT‐C*_n_*) with the alkyl chain (‐C*_n_*) length of *n*≥ 5.^[^ ^18^, ^19^, ^20^, ^21^, ^22^, ^23^, ^24^ ^]^ The molecules prefer to align unidirectionally *side‐by‐side* to each other, thus forming polar monomolecular layers. The eventual high layered crystallinity allows us to produce very large‐area and extremely uniform single‐crystal thin films by a simple solution‐coating process under ambient conditions.^[^ ^25^, ^26^, ^27^ ^]^ It was demonstrated that the alkyl chains take crucial roles in achieving high layered crystallinity, where the self‐organizing nature of the π‐cores and the alkyl chains are coupled and enhance each other synergistically. The feature is observed in several compounds of π‐core–alkyl‐chain linked molecular system.^[^ ^28^, ^29^, ^30^, ^31^, ^32^, ^33^, ^34^, ^35^ ^]^ They involve well‐aligned 2D π‐core layers that exhibit excellent organic field‐effect transistor (OFET) characteristics, providing a promising component for 2D molecular materials and devices.^[^ ^36^, ^37^, ^38^, ^39^ ^]^

The polar monomolecular layers are stacked alternately to each other in an antiparallel fashion, which eventually forms bilayer‐type herringbone (*b*‐LHB) packing in the compounds reported thus far. This antipolar‐type interlayer stacking causes total polarity to be canceled out for whole crystals. If the interlayer stacking arrangements can be controlled, further types of materials and device functions are expected, such as piezoelectric, electro‐optic, and photovoltaic effects, in which the uniaxial molecular orientation may amplify the original electric and/or optical responses of the unsymmetric molecules with use of the exceptionally uniform layered‐crystalline structures. However, the interlayer stacking has never been tunable in the 2D molecular materials.

**Figure 1:**
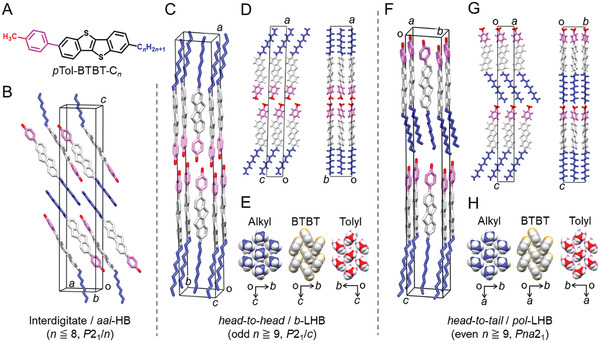
Crystal packings of a series of *p*Tol‐BTBT‐C*_n_*. A) Chemical structure of *_p_* Tol‐BTBT‐C*_n_*. Crystal packings of *p*Tol‐BTBT‐C*_n_* for B) *n* = 8, C) *n* = 9, and F) *n* = 10. Crystal packings projected along the *a‐c* plane and *a‐b* plane for D) *n* = 9 and G) *n* = 10. Herringbone packings of alkyl chains, BTBT cores, and tolyl groups for E) *n* = 9 and H) *n* = 10 in monomolecular layers. The BTBT moiety is shown by the color of white whereas alkyl chains, phenyl groups, and end methyl groups by blue, pink, and red.

In this study, we report that the polar/antipolar type interlayer stacking can be controlled in the newly designed 2D materials, by taking advantage of the length‐variable nature of alkyl chains. We first assume that the antipolar‐type *b*‐LHB packing should be stabilized by relatively strong interlayer *head‐to‐head* interaction. Hence, we converted the phenyl group of Ph‐BTBT‐C*_n_* into *para*‐tolyl (*p*Tol) group, to develop a series of *p*Tol‐BTBT‐C*_n_*, shown in **Figure** **1**A. We found that the methyl substitutions effectively suppress the *head‐to‐head* interaction with retaining the well‐aligned herringbone array of polar monomolecular layers at *n*≥ 9. As a result, drastic alkyl‐parity dependent alternation of polar/antipolar type packing emerges because of the competition between *head‐to‐head* and *head‐to‐tail* interactions in the crystals. Herein, we present all the crystal structure analysis of the series of *p*Tol‐BTBT‐C*_n_* up to *n* = 5 to 14, and discuss the origin of the drastic odd‐even effect on the polar/antipolar alternation of interlayer stacking at *n*≥ 9. Then we show highly homogeneous thin film formation, optical second harmonic generation (SHG) response, and also single‐crystal OFET characteristics.

## 2 Results and Discussion

### 2.1 Chain Length Dependence of Crystal Structures

We developed and synthesized a series of *p*Tol‐BTBT‐C*_n_* substituted with various alkyl chain lengths (*n* = 5−14) by a synthetic route as described in Figure S1 (Supporting Information). Full crystal structure analyses were successfully conducted for all the compounds, the results of which are presented in Figure 1, Figures S2−S7, and Table S1 (Supporting Information). The obtained packing motifs of all the compounds are composed of molecular alignments with long axes roughly parallel to each other but can be classified into three categories, depending on the alkyl chain length, as summarized in Figure 1. The first motif is observed in short‐chain compounds with *n* = 5−8 (Figure 1B) with a monoclinic space group of *P*2_1_/*c* or *P*2_1_/*n*, where the independent molecular layers are not formed. The adjacent molecules are aligned antiparallel to each other, and the alkyl chains are interdigitated between the neighboring π‐core layers under inversion symmetry. We call the motif as antiparallel alkyl‐interdigitated herringbone (*aai*‐HB) packing. Such a unique packing motif seems to be similar to those of Ph‐BTBT‐C_4_ and other compounds.^[^ ^19^, ^35^ ^]^ In contrast, the long‐chain compounds with *n* = 9−14 exhibit high‐layered crystallinity with forming independent monomolecular layers. The second motif is the *b*‐LHB‐type packing observed in the case of long‐chain and odd‐*n* compounds with *n* = 9, 11, and 13 (Figure 1C−E), as is similar to the case of Ph‐BTBT‐C*_n_* with *n* ≥ 5.^[^ ^19^ ^]^ The polar monomolecular layers are formed and stacked alternately by the *head‐to‐head* and *tail‐to‐tail* molecular alignment with showing inversion symmetry (space group of *P*2_1_/*c*). The final arrangement is observed in the case of long‐chain and even‐*n* compounds with *n* = 10, 12, and 14 (Figure 1F−H), forming unique polar crystals without inversion symmetry (orthorhombic space group of *Pna*2_1_), where the respective polar monomolecular layers are stacked uniformly by *head‐to‐tail* molecular alignments along *c* axis. We refer to this unique crystal structure as polar‐type LHB (*pol*‐LHB) packing.

A comparison of the overall *n*‐dependence of the packing motifs between *p*Tol‐BTBT‐C*_n_* and Ph‐BTBT‐C*_n_* demonstrates that the methyl substitution partially hindered the emergence of *b*‐LHB packing. First, we notice that independent polar monomolecular layers are formed at *n*≥ 9 in *p*Tol‐BTBT‐C*_n_*, while they are observed at shorter chain length of *n*≥ 5 in Ph‐BTBT‐C*_n_* by forming the *b*‐LHB packing.^[^ ^19^ ^]^ It means that a larger interchain interaction with longer alkyl chain length is necessary to stabilize the independent monomolecular layer in *p*Tol‐BTBT‐C*_n_*. Second, we notice that the intralayer molecular packing arrangements are very similar to each other for the crystals of *p*Tol‐BTBT‐C*_n_*, irrespective of the parity *n*, at 9 ≤*n* ≤14, as presented in Figure S6 (Supporting Information). Such isomorphous nature of the monomolecular layer also contrasts with the seemingly similar but slight *n*‐dependent variation of intralayer packing motifs in Ph‐BTBT‐C*_n_* at *n*≥ 5. Both of the above features could be attributed to the suppression of *head‐to‐head* intermolecular interaction between tolyl groups, which is supported by the intermolecular interaction calculation as shown in the next subsection.

### 2.2 Origin of the Odd–Even Alternation of Polar/Antipolar Interlayer Stacking

**Figure 2:**
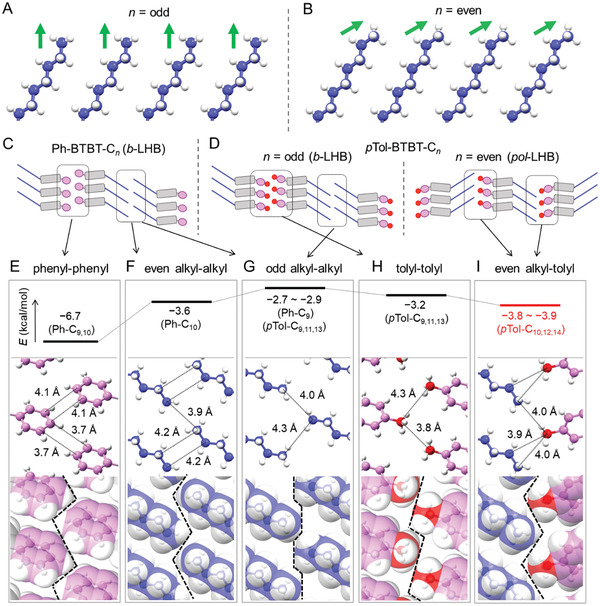
Interlayer arrangements in single crystals. The terminal C─C bond orientation of alkyl chains in *p*Tol‐BTBT‐C*_n_* crystals for A) odd *n* and B) even *n*. Packing diagrams of C) Ph‐BTBT‐C*_n_* and D) *p*Tol‐BTBT‐C*_n_* projected along the axis parallel to the π‐stacking layers, and their interlayer *end‐to‐end* interaction energies (top) and arrangements around E) phenyl‐phenyl, F) even alkyl‐alkyl, G) odd alkyl‐alkyl, H) tolyl‐tolyl, I) even alkyl‐tolyl groups depicted by ball‐and‐stick views (middle) and by space‐filling views (bottom). The detail of the DFT calculation is shown in Supporting Information. E–I) is highlighted at the dotted square in (C) and (D). Green arrows show the terminal C─C bond orientations. The black dotted lines show the connection of the carbon atoms less than 4.5 Å in the next layer. The black dashed lines schematically show the *layer‐by‐layer* boundary.

To disclose the origin of the alkyl‐parity controlled switching of polar/antipolar interlayer stacking arrangements, we conducted dispersion‐corrected density functional theory (DFT) calculations of intermolecular interactions based on the atomic coordinates of the obtained packing motifs. The calculated intermolecular interaction energy takes all electronic interaction into account. We here focus on the intermolecular *end‐to‐end* interaction between rodlike molecules at the interlayer contacts, as depicted in **Figure** **2**. This is because the terminal C─C bond orientation of the alkyl substituents is distinct between the odd‐ and even‐*n* crystals in *p*Tol‐BTBT‐C*_n_* at *n* = 9−14 (Figure 2A,B), as is expected from the all‐*trans* alkyl chains, which should directly affect the interlayer interaction. Figure 2C–I, Figure S8, and Table S2 (Supporting Information) summarize the calculated results.

Prior to the arguments on the results, we mention that the dispersion force is the dominant attractive intermolecular interaction for both π‐cores and alkyl chains in such single‐component organic semiconductors (OSCs).^[^ ^20^ ^]^ As the strength of the dispersion forces depends mainly on the atomic polarizability and atom‐atom distance, the distance between carbon atoms that have much higher polarizability than hydrogen atoms is more significant for achieving stronger dispersion interactions. Namely, when the carbon atoms become closer to each other, the intermolecular interactions become stronger.

In the calculated results, we first notice that the phenyl‐phenyl interaction in Ph‐BTBT‐C*_n_* is the strongest (−6.7 kcal mol^−1^) among the other *end‐to‐end* contacts, as shown in Figure 2E–I. This is the reason why the *b*‐LHB packing, composed of phenyl‐phenyl (or *head‐to‐head*) interaction, is stabilized in Ph‐BTBT‐C*_n_*. In contrast, the *end‐to‐end* interactions between alkyl chains depend on the terminal C−C bond orientation in Ph‐BTBT‐C*_n_*; the interaction is larger at *n* = 10 than that at *n* = 9 (−3.6 and −2.7 kcal mol^−1^, respectively) as shown in Figure 2F,G. The difference is ascribed to the fact that the end carbon atom at *n* = 10 (*n* = 9) becomes closer (farther) to the layer surface when the terminal C−C bond orientation is more (less) inclined to the layer normal. However, the variation is too small to affect the whole packing motif but is observed only as an odd‐even oscillation of the liquid‐crystalline (LC) transition entropy in Ph‐BTBT‐C*_n_*.^[^ ^19^ ^]^

Second, we can confirm that the end‐methyl substitution of phenyl group in Ph‐BTBT‐C*_n_* effectively suppresses the *head‐to‐head* (tolyl‐tolyl) interaction (−3.2 kcal mol^−1^) in *p*Tol‐BTBT‐C*_n_*, as shown in Figure 2E,H. The result is associated with the fact that the end‐methyl carbon in *p*Tol group is surrounded by three bonded hydrogens, which leads to suppress the short‐contact between carbon atoms through the steric hindrance (or exchange repulsion) of methyl groups. The result is consistent with the emergence of independent monomolecular layers at longer chain length of *n*≥ 9 in *p*Tol‐BTBT‐C*_n_*, as compared to the case of Ph‐BTBT‐C*_n_* at *n*≥ 5. It means that the larger interchain interaction with longer alkyl chain length is necessary to stabilize the independent monomolecular layer in *p*Tol‐BTBT‐C*_n_*. Conversely, the interlayer *head‐to‐head* (phenyl‐phenyl) interaction should contribute to stabilize the *b*‐LHB packing in Ph‐BTBT‐C*_n_*. The result is also consistent with the increase in solubility of *p*Tol‐BTBT‐C*_n_* as compared to that of Ph‐BTBT‐C*_n_*, as discussed later.

**Figure 3:**
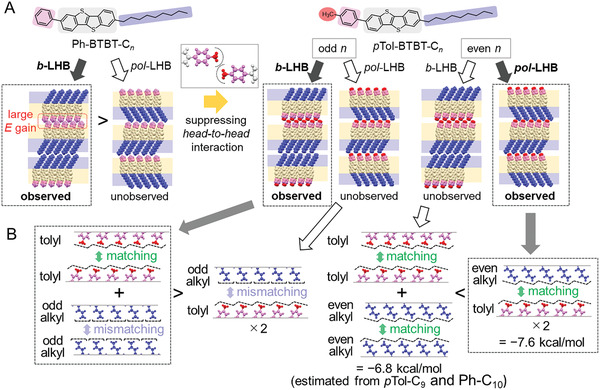
Schematics of the possible *layer‐by‐layer* stacking combinations. A) Schematics of the Ph‐BTBT‐C*_n_* (left) and *p*Tol‐BTBT‐C*_n_* (right). The orange and blue areas show π‐core layers and alkyl chain layers, respectively. B) Schematics of the affinities in the layer surface for tolyl groups, odd‐*n* alkyl chains, and even‐*n* alkyl chains. The black dashed lines schematically show the boundary of the space‐filling surface.

**Figure 4:**
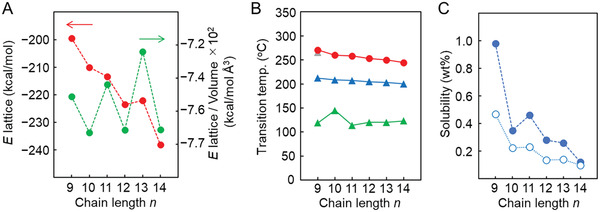
Changes in lattice energies, solubilities, and phase‐transition temperature. A) Changes in lattice energies. Red ●: calculated from the crystal structure using electronic structure calculation package of Quantum Espresso and green ●: lattice energies per volume. B) Changes in phase‐transition temperature of *p*Tol‐BTBT‐C*_n_* determined by DSC measurements. Green ▲: crystal to liquid‐crystal phase transition, blue ▲ and gray ▲: liquid‐crystal to liquid crystal phase transition, and red ●: melting point. C) Solubility changes in chlorobenzene at 20 °C determined by the concentration of saturated solution. Blue ●: *p*Tol‐BTBT‐C*_n_* and white ○: Ph‐BTBT‐C*_n_*.

Finally, we discuss that alkyl‐parity switching of polar/antipolar packing arrangements as observed in *p*Tol‐BTBT‐C*_n_* should be caused by the odd‐even alternation of terminal C─C bond orientation. It is obvious that the effect comes to appearance when the *head‐to‐head* interaction is suppressed. Among the possible *end‐to‐end* combinations (tolyl–tolyl, tolyl–alkyl, and alkyl–alkyl), as shown in **Figure** **3**, the alkyl–tolyl combination is relatively more stable at even *n*, (−3.8 kcal mol^−1^) where the terminal C─C bond orientation of substituted alkyl chain is more inclined than the layer normal. At the interlayer contact, one of the terminal hydrogen atoms of the alkyl chains protrudes into the hollow of the counter *p*Tol layer surface by avoiding the steric hindrance (exchange‐repulsion) of hydrogen atoms. The situation allows the end‐methyl carbon atoms to get closer position, which eventually enhance the *end‐to‐end* interaction, as shown in Figures 2I and 3B. In contrast, the terminal C─C bond orientations for both the *p*Tol and alkyl groups were approximately parallel to the layer normal at odd *n*. The *end‐to‐end* interaction between tolyl and odd‐*n* alkyl should be much weaker than that between tolyl and even‐*n* alkyl, although it is not possible to compare those interaction directly because of the lack of the actual crystallographic data. Eventually, tolyl–tolyl and alkyl–alkyl combinations are selected to form the *b*‐LHB packing in the actual odd‐*n* crystals. However, the stability of the *b*‐LHB packing in the odd‐*n* crystals may be quite limited compared to that of Ph‐BTBT‐C*_n_*. **Figure** **4**A presents the calculated lattice energy per unit volume evaluated from the single‐crystal packing data. The result indicates that the *pol*‐LHB packing in the even‐*n* crystals is relatively more stable than the *b*‐LHB packing in the odd‐*n* crystals. From the above arguments, we conclude that the alkyl‐parity switching of polar/antipolar interlayer stacking arrangements is triggered by the odd‐even alternation of alkyl‐terminal C─C bond orientations.

### 2.3 Thermal Analysis and Solubility

The odd‐even oscillations are more or less observed in thermal properties and solubilities of *p*Tol‐BTBT‐C*_n_* (*n* = 5−14). The thermal analysis using differential scanning calorimetry shows three endothermic peaks at high temperatures for all the compounds, irrespective of the parity of *n* (Figures S9 and S10, and Table S3, Supporting Information). The observed features were similar to each other in odd‐ and even‐*n* compounds and also close to those of Ph‐BTBT‐C*_n_*.^[^ ^19^ ^]^ The first peaks observed in the range of 120−150 °C can be ascribed to the LC transition to the smectic *E* phases; the observed powder X‐ray diffraction (XRD) profiles for *n* = 9 and 10 compounds seem to be common with those for Ph‐BTBT‐C*_n_* (Figure S11, Supporting Information). Nonetheless, a slight odd‐even oscillation in the LC transition temperature is observed as shown in Figure 4B. The features could be ascribed to the fact that the intralayer molecular arrangements that dominate the lattice energy are common for odd‐ and even‐*n* crystals of *p*Tol‐BTBT‐C*_n_*, whereas the crystals exhibit drastic odd–even alternation of interlayer stacking arrangements.

The solubility of *p*Tol‐BTBT‐C*_n_* at *n* = 5−14 is two times higher than that of Ph‐BTBT‐C*_n_*, and decreases as the chain length increases, with showing a slight odd–even oscillation, as shown in Figure 4C and Table S4 (Supporting Information). The observed oscillation in *p*Tol‐BTBT‐C*_n_* is more pronounced than that in Ph‐BTBT‐C*_n_*. The features in thermal properties and solubilities are consistent with the variations in lattice energies obtained by quantum chemical intermolecular interaction calculations, as shown in Figure 4A.

### 2.4 On the Polar Nature of Monomolecular Layers and Whole Crystals

We investigated the electric dipole moment of molecules and their sum for the monomolecular layers and for whole crystals by using time dependent DFT calculations (B3LYP/6‐31G(d)). Transition dipole moments are calculated to be finite (retained) with large component along the molecular long axis in isolated unsymmetric molecules of *p*Tol‐BTBT‐C*_n_* at *n* = 11 and 12, as presented in Figure S12 (Supporting Information), respectively. The monomolecular layer formed by the uniaxial orientation of the molecular long axis naturally possesses the polarization along normal to the layer plane. Additionally, intralayer polarization is retained along the direction parallel to the glide plane (or along *c*‐axis for odd‐*n* and along *a*‐axis for even‐*n*), which is parallel to the slipped‐parallel directions in the *b*‐LHB and *pol*‐LHB packings of odd‐ and even‐*n* crystals, respectively. In contrast, the polarization is vanished along *b*‐axis, as depicted in Figure S12C (Supporting Information).

The unit cell length along the interlayer stacking axis is doubled of the monomolecular layer thickness for both odd‐ and even‐*n* crystals. As the *b*‐LHB packing possesses inversion symmetry with a space group of *P*2_1_/*c*, the odd‐*n* crystals are nonpolar in any 3D directions. It means that the polarization of the monomolecular layer is coupled antiparallelly by the bilayer formation. In contrast, adjacent monomolecular layers in the *pol*‐LHB packing are related to each other by the *n*‐glide symmetry with a space group of *Pna*2_1_, with regard to the glide plane that is perpendicular to the *a*‐axis. The feature leads to the emergence of polarization along the interlayer stacking direction for whole crystals, whereas the intralayer polarizations form antiparallel coupling between the adjacent monomolecular layers. Thus, it is reasonable to call the odd‐ and even‐*n* crystals as antipolar and polar‐layered organic semiconductors, respectively.

### 2.5 Solution‐Processed Single‐Crystal Thin Films

**Figure 5:**
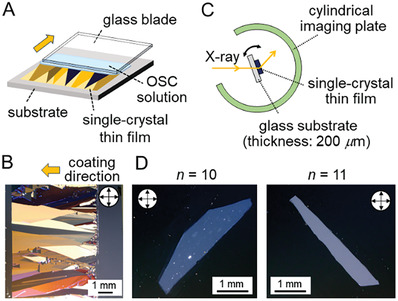
The single‐crystal thin‐film fabrication by the blade‐coating technique and experimental set up for the XRD measurement. A) Schematic of a blade‐coating method. B) A crossed‐Nicols polarized optical microscope image of the blade‐coated single‐crystal thin film of *p*Tol‐BTBT‐C_10_. As the film is composed of several single crystal domains, we used a single domain after trimming other crystal domains away for the XRD measurements. C) Schematic of an experimental setup for the XRD measurement using single‐crystal thin film. D) Crossed‐Nicols polarized micrographs of isolated single‐crystal thin film of *p*Tol‐BTBT‐C_10_ (left) and *p*Tol‐BTBT‐C_11_ (right) on thin‐glass substrate used for the XRD measurement, respectively. The film thicknesses are 25 nm (5 unit‐cell layer) in both cases.

A blade‐coating technique was used to manufacture single‐crystal thin films of *p*Tol‐BTBT‐C*_n_* (*n* = 5−14) on silicon substrates with 100 nm‐thick silicon dioxide layers, as schematically depicted in **Figure** **5**A. Films with large single‐crystal domains with high thickness uniformity were obtained for the compounds at *n* = 9−14, as exhibited in Figure 5B,D. From the XRD measurements of the films at *n* = 10 and 11 shown in Figure 5C,D and Figure S13 (Supporting Information), we confirmed that the unit cell parameters of both films are consistent with those of the bulk crystals, as listed in **Table** **1**. In addition, the out‐of‐plane diffractions obey the extinction rules, as expected from the respective crystal lattices, as shown in Figures S14 and S15 (Supporting Information). The results demonstrate that the drastic parity‐dependent polar/antipolar alternation of the crystal packing was retained in the single‐crystal thin films. Nevertheless, we could not determine the up‐or‐down crystallographic orientation of the polar single‐crystal films by the observed diffraction peaks, because sufficient intensity to assign the absolute orientation of the crystal lattice was not obtained because of the fairly thin thickness of the films.

**Table 1:** Lattice constants of *p*Tol‐BTBT‐C*_n_* at *n* = 10 and 11 in bulk single crystals and single‐crystal thin films.

Lattice constants	*p*Tol‐BTBT‐C_10_		*p*Tol‐BTBT‐C_11_	
	bulk	thin film	bulk	thin film
*a* (Å)	5.929	5.917	59.180	59.523
*b* (Å)	7.766	7.784	7.815	7.792
*c* (Å)	56.223	56.520	5.915	5.883
*α* (deg.)	90	90.18	90	90.18
*β* (deg.)	90	90.01	91.97	93.38
*γ* (deg.)	90	89.99	90	90.29

In contrast to the high film formability at *n* = 9−14, it was much more difficult to obtain highly uniform thin films in the compounds at *n* = 5−8. It is clear that such a difference in film formability is ascribed to the difference in layered crystallinity between the LHB packing (*n* ≥ 9) composed of nearly independent monomolecular layers and the *aai*‐HB packing with interlayer alkyl interdigitation (*n* = 5−8).

**Figure 6:**
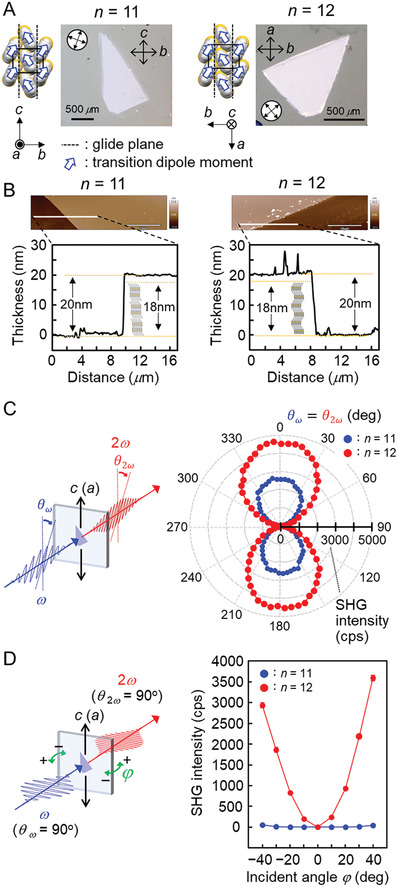
Optical SHG measurements on the single‐crystal thin‐film. A) The arrangement of the glide plane and the transition dipole moment in the respective monomolecular layer, and a crossed‐Nicols polarized optical microscope image of the blade‐coated single‐crystal thin film of *p*Tol‐BTBT‐C_11_ (left) and *p*Tol‐BTBT‐C_12_ (right). B) The film thickness of single‐crystal thin‐film of *p*Tol‐BTBT‐C_11_ (left) and *p*Tol‐BTBT‐C_12_ (right) measured by AFM. C) Schematic of an experimental setup for optical SHG measurements at normal incidence (left), and the polarization angle dependence of the SHG intensity for *p*Tol‐BTBT‐C_11_ (●) and *p*Tol‐BTBT‐C_12_ (●) (right). D) Schematic of an experimental setup for optical SHG measurements at oblique incidence (left), and the incident angle dependence of the SHG intensity for *p*Tol‐BTBT‐C_11_ (●) and *p*Tol‐BTBT‐C_12_ (●) (right).

For optical SHG measurements, we also fabricated single‐crystal thin films of *p*Tol‐BTBT‐C*_n_* at *n* = 11 and 12 on transparent thin glass substrates, as presented in **Figure** **6**A,B. We measured polarization angle dependence of polarized optical absorption spectra for the films, the results of which are shown in Figures S16C and S17C (Supporting Information). The observed optical anisotropy is consistent with that of Ph‐BTBT‐C*_n_*. The polarization angles at which the absorption become maxima should correspond to the *c*‐axis in the antipolar single‐crystal film at *n* = 11 and to the *a*‐axis in the polar single‐crystal film at *n* = 12, as shown in Figures S16 and S17 (Supporting Information), respectively.

### 2.6 Optical SHG Measurements of Single‐Crystal Thin Films

We conducted optical SHG measurements for the *p*Tol‐BTBT‐C_*n*_ single‐crystal thin films on transparent substrates. Optical SHG is particularly useful for detecting polar states because, in the leading order, SHG occurs only in non‐centrosymmetric media.^[^ ^40^, ^41^ ^]^ As illustrated in Figure 6C,D, we performed two different kinds of angle‐dependent SHG measurements in transmission geometry to investigate polar/antipolar interlayer stacking. Figure 6C shows the rotational anisotropy of the SHG intensity, which was obtained at normal incidence by projecting the component of the SHG light *θ*_2ω_ oriented parallel to the polarization *θ*_ω_ of the incident fundamental light while *θ*_ω_ = *θ*_2ω_ was rotated over 360°. The variation of the SHG intensity exhibited a gourd‐shaped profile for both *n* = 11 (*b*‐LHB packing) and *n* = 12 (*pol*‐LHB packing). The SHG intensity shows maxima at *θ*_ω_ = *θ*_2ω_ = 0° and 180° (namely, along *c*‐axis for *n* = 11 and along *a*‐axis for *n* = 12), where the polarization is retained for monomolecular layers but should be canceled out for whole crystals in both cases (Figure S12, Supporting Information). In contrast, the SHG signal is considerably suppressed at *θ*_ω_ = *θ*_2ω_ = 90° and 270° (namely along *b*‐axis), at which the polarization of monomolecular layers is vanished for both films.

Figure 6D shows the incident angle dependence of the SHG intensity. The measurements were conducted with fixed polarization angles (*θ*_ω_ = *θ*_2ω_ = 90°), at which the in‐plane SHG signals are not observed at normal incidence (Figure 6C). An increase of the incident angle leads to a pronounced increase in the SHG intensity for *n* = 12, but much smaller change is observed for *n* = 11. This cannot be explained by the surface SHG contribution due to the inversion symmetry breaking by the discontinuity at the surface,^[^ ^40^ ^]^ but by the presence (absence) of out‐of‐plane polarity for *n* = 12 (*n* = 11). The result demonstrates that the *pol*‐LHB packing exhibits strong SHG response even in the extremely thin film due to the presence of out‐of‐plane polarity. The finding offers possibilities for the applications such as photo‐responsive semiconductor devices.

We then go back to the observation of in‐plane SHG signal at normal incidence (Figure 6C), which should disappear when we consider the whole crystals for both *n* = 11 and 12. It is most probable that the SHG signal could be ascribed to the presence of an excess monomolecular layer. We consider that the excess layer should be formed more easily in *p*Tol‐BTBT‐C_*n*_ than in Ph‐BTBT‐C_*n*_, because of the suppression of *head‐to‐head* intermolecular interaction as described before. Actually, the films used for the SHG measurements have thickness that indicates the presence of an additional monomolecular layer, as seen in atomic force microscope (AFM) images in Figure 6B. We note that such halfway step height associated with the monomolecular layer is not observed in single‐crystal thin‐films of Ph‐BTBT‐C_10_ fabricated using the same blade‐coating technique.^[^ ^25^ ^]^ The feature is ascribable to the larger *head‐to‐head* affinity between the Ph‐BTBT‐C_10_ molecules.

### 2.7 Field‐Effect Transistor Characteristics

**Figure 7:**
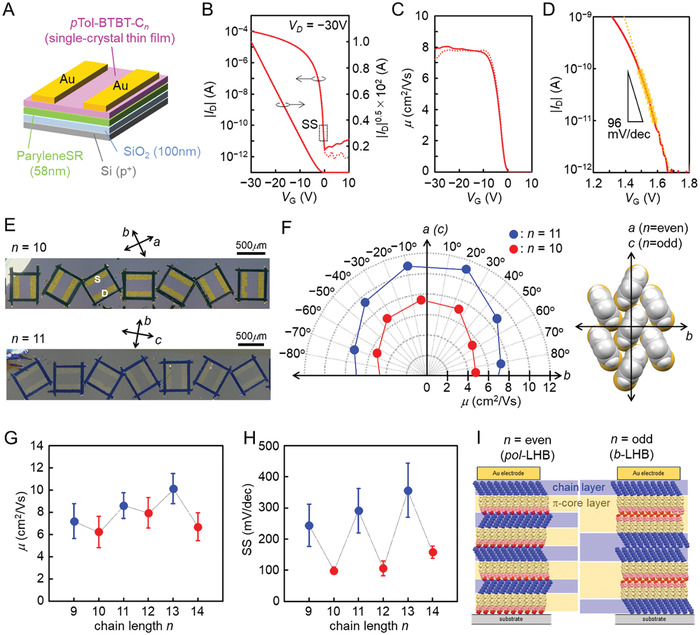
Single‐crystal OFET device performance. A) Schematic of the bottom‐gate/top‐contact (BGBC) OFET. B) typical transfer characteristics for *p*Tol‐BTBT‐C_10_ single crystal measured at *V*_D_ = −30 V. C) The plot of mobility as a function of *V*_G_. D) Transfer characteristics in the switching region. E) Optical microscope images of OFET devices for *p*Tol‐BTBT‐C_10_ (top) and *p*Tol‐BTBT‐C_11_ (bottom) using the measurements of mobility anisotropy. The direction of the crystallographic axis in the film was determined by polarized absorption measurement as described in Figure S21 (Supporting Information). F) Anisotropy of the mobility (left) for *p*Tol‐BTBT‐C_10_ (●) and *p*Tol‐BTBT‐C_11_ (●), and corresponding crystallographic axis in herringbone packing (right). G) Alkyl chain length dependence of the mobility changes at the saturation region. H) Alkyl chain length dependence of the SS values at the saturation region. I) Schematic of the cross‐sectional molecular arrangements of *b*‐LHB packing and *pol*‐LHB packing in BGTC device.

To investigate the impact of the distinct odd–even effect on carrier transport characteristics, we produced standard bottom‐gate/top‐contact (BGTC)‐type single‐crystal OFETs based on *p*Tol‐BTBT‐C_*n*_ with different alkyl chain lengths (*n* = 9−14). We employed two kinds of gate dielectrics: the parylene‐coated SiO_2_ layers and the sole SiO_2_ layers. Typical device characteristics and layer number dependence of the mobilities are shown in **Figure** **7**; Figure S18 and Table S6 (Supporting Information) for the former and in Figures S19 and S20 (Supporting Information) for the latter. We confirmed by the out‐of‐plane XRD measurements that the crystal packings of the OSC layers are the same as the bulk crystals, as presented in Figure S21 (Supporting Information). We found that the single‐crystal OFETs composed of sole SiO_2_ gate dielectric layer exhibit high average mobility of ≈10 cm^2^ V^‐1^s^−1^, but suffer from a large threshold voltage (*V*_th_ ≈−20 V) and large hysteresis. In contrast, the single‐crystal OFETs composed of parylene‐coated SiO_2_ gate dielectric layer show improved transfer characteristics with extremely sharp switching performance, no hysteresis, and low *V*_th_ (≈0 V), as shown in Figure 7A–D. It is most probable that the large Vth and hysteresis as observed in the former devise could be ascribed to the interfacial carrier traps at high density that exist between the OSC and untreated SiO_2_ layer.^[^ ^42^, ^43^, ^44^ ^]^ Thus we used the results of the latter devices to discuss intrinsic device characteristics.

We also fabricated single‐crystal OFET based on *p*Tol‐BTBT‐C_*n*_ at *n* = 8 that forms *aai*‐HB packing in the bulk crystal. The device shows relatively lower mobility of ≈1 cm^2^ V^‐1^s^−1^, as compared to the devices based on the compounds at *n* = 9−14. It is clear that the lower mobility should be attributed to the difference in the molecular packing.

We estimated the mobility anisotropy in the single‐crystal OFETs composed of *p*Tol‐BTBT‐C_*n*_ at *n* = 10 and 11, the results of which are shown in Figure 7E–G. We used polarized optical reflection measurements for determining the crystallographic orientation of the channel OSCs, the detail of which is depicted in Figure S22 (Supporting Information). The highest mobility is observed along the *a*‐ (or *c*‐) axis (i.e., along the slipped parallel contact), and the lowest mobility is observed along the *b*‐axis (i.e., along the *T*‐shaped contact). The ratio of the mobility between *a*‐ (or *c*‐) axis and *b*‐axis was ≈3:2, which is well consistent with the other BTBT‐based single‐crystal OFETs having *b*‐LHB packings.^[^ ^39^ ^]^ The results also indicate that the in‐plane anisotropy of the mobility is not affected by the interlayer stacking arrangements.

We summarize the *n*‐dependence of device characteristics in Figure 7G,H. The mobility is high enough in single‐crystal OFETs of *p*Tol‐BTBT‐C_*n*_’s in both odd and even cases. We also found that the odd‐even oscillation was observed slightly in the mobility values and relatively notably in the subthreshold swing (SS) values. The mobility of odd‐*n* crystals (*b*‐LHB) is slightly larger than that of even‐*n* crystals (*pol*‐LHB). In contrast, the SS value at even *n* is much smaller (or more excellent) than that at odd *n*. The obtained results are quite intriguing, although it might not be straightforward to understand the origin of the notable odd‐even oscillation in the SS values. We consider that one of the reasons for the observation should be ascribed to the difference in the *layer‐by‐layer* stacking arrangements, and more concretely to the difference in the thickness of the inert alkyl chain layer. The thickness of the alkyl chain layer in the *pol*‐LHB structure is about half of that in the *b*‐LHB packings (see Figure 7I). We consider that the thermal diffusion of the carrier from the electrode to the channel layer should be more efficient due to the thin inert layer thickness in the *pol*‐LHB packing, eventually providing the more excellent SS values. On the other hand, we also consider that the slightly higher mobility observed in the *b*‐LHB packing should be associated with the thicker π‐core layer, which could be effective for decreasing phonon scattering. Although the BGTC device is the standard OFET structure, it is still challenging to reveal and understand the precise correlation between device characteristics and molecular arrangements in the OSC layers. We believe that the systematic control of the molecular arrangements in the highly layered‐crystalline OSCs should be quite useful to understand and realize the ideal carrier transport in single‐crystal OFETs.

## 3 Conclusion

In summary, we successfully developed a series of highly layered‐crystalline organic semiconductors of *p*Tol‐BTBT‐C_*n*_ whose solid‐state polarity is switchable by the parity of substituted alkyl chain length *n*, in both bulk single crystals and crystalline thin films. The even‐*n* compounds (*n* = 10, 12, and 14) form unique *pol*‐LHB packing composed of unidirectionally aligned polar molecules over the entire intralayer and interlayer arrangements, whereas the odd‐*n* compounds (*n* = 9, 11, and 13) present usual *b*‐LHB packing comprising alternating antipolar‐type interlayer stacking. The emergence of the notable odd–even effects at *n* = 9−14 is caused by the introduction of end methyl group, which effectively suppresses the *head‐to‐head* interactions and balances the intermolecular *end‐to‐end* affinity. It was demonstrated by dispersion‐corrected DFT calculations of intermolecular interactions that the emergence of the alkyl‐parity controlled polar/antipolar switching is caused by the odd‐even alternation of alkyl‐chain‐terminal C─C bond orientations which controls the interlayer coupling. The LC transition temperature and the solubility exhibit slight odd–even oscillations, which are consistent with the isomorphous nature of the intralayer packing and the drastic variation of weak interlayer stacking accompanied by the change in coupling energy. A single‐crystal thin‐film of a series of *p*Tol‐BTBT‐C_*n*_ could be readily fabricated by the blade‐coating technique at room temperature. The obtained films exhibited strong optical SHG signals, particularly in thin‐films with the *pol*‐LHB packing, due to the polarity switching of the crystals based on the parity of alkyl chains. The intrinsic device mobility of *p*Tol‐BTBT‐C_*n*_ is as high as 10 cm^2^ V^‐1^s^−1^ with excellent sharp switching in the BGTC‐type single‐crystalline OFETs, and show notable odd–even oscillations. The findings pave the way for creating polarity‐controlled optoelectronic materials and devices based on various functional alkylated compounds. The packing evolution from *n* = 5,6,7,8 (composed of antiparallel alkyl‐interdigitated packing between layers) to *n* ≥ 9 (composed of independent polar monomolecular layers) in *p*Tol‐BTBT‐C_*n*_ allows us to understand important aspect of the role of alkyl chains in obtaining the highly layered‐crystalline OSCs, particularly by a comparison with the similar packing evolution in Ph‐BTBT‐C_*n*_.

## 4 Experimental Section

### Materials Synthesis

All *p*Tol‐BTBT‐C*_n_* (*n* = 5−14) compounds were synthesized via the Suzuki coupling of the corresponding Br‐BTBT‐C*_n_* and *para*‐tolylboronic acid. Corresponding Br‐BTBT‐C*_n_* were synthesized by the reported procedure.^[^ ^19^ ^]^ The experimental procedures were summarized in Supporting Information.

### Crystal Structure Analyses

Single crystals of *p*Tol‐BTBT‐C*_n_* for structural analysis were obtained by recrystallization from a saturated solution in anisole at room temperature. The crystals obtained in the solution were carefully transferred to a mounting apparatus directly for structural analysis (LithoLoops, Protein Wave Corp.), as shown in Figure S2 (Supporting Information). Single‐crystal XRD measurements for *n* = 5−10, 12, and 14 were performed using a Rigaku AFC10 four‐circle diffractometer equipped with a Piratus 200K hybrid pixel detector. For *n* = 11 and 13, the measurements were performed using a Rigaku cylindrical imaging plate system, and a monochromatized synchrotron radiated X‐ray beam at beamline BL‐8A of the KEK (High Energy Accelerator Research Organization) Photon Factory. Data correction and reduction were conducted using the CrysAlisPro (Rigaku Corp.) and Rapid‐AUTO software packages (Rigaku Corp.). Refinement was performed using the Olex2^[^ ^45^ ^]^ and CrystalStructure software packages (Rigaku Corp.). Each initial structure was solved by direct methods using either of SIR92,^[^ ^46^ ^]^ SIR2004,^[^ ^47^ ^]^ and SIR2008^[^ ^48^ ^]^ programs and was refined via the full‐matrix least‐squares method using SHELXL^[^ ^49^ ^]^ by applying anisotropic temperature factors for nonhydrogen atoms. Hydrogen atoms were placed at geometrically calculated positions. The obtained crystallographic parameters are listed in Table S1 (Supporting Information).

### Density Functional Theory Calculations

Intermolecular interactions between a *p*Tol‐BTBT‐C*_n_* molecule and neighboring molecules (with atom‐atom contact less than 8 Å) were calculated using the crystal geometries, as shown in Figure S6 (Supporting Information), to compare the interlayer and intralayer interactions. The Gaussian 16 program^[^ ^50^ ^]^ was used for the DFT calculations. Intermolecular interaction energies were calculated at the PBE/6‐311G** level using Grimme's D3BJ dispersion correction.^[^ ^51^ ^]^ Basis set superposition error (BSSE) was corrected using the counterpoise method.^[^ ^52^, ^53^ ^]^ The sum of the interaction energies with the neighboring molecules is listed in Table S2 (Supporting Information).

The Quantum Espresso^[^ ^54^, ^55^ ^]^ was used for the DFT calculations of the energies of crystals and isolated molecules to evaluate the lattice energies. The energies were calculated using the PBE functional and Grimme's D3BJ dispersion correction.^[^ ^51^ ^]^ The cutoff energies of plane wave basis set and charge density were 36 Ry and 324 Ry, respectively. Each molecule was isolated by the cubic unit cell with the edge of 35 Å. The experimental cell parameters were fixed during the optimization of molecules in crystals. The lattice energies (*E*_lattice_) were obtained according to the following equation,

(1) $$E_{\mathrm{lattice}}=E_{\mathrm{cryst}}-4E_{\mathrm{mono}}$$

where *E*_cryst_ is the energy of the unit cell of crystal after geometry optimization, which contains four molecules, and *E*_mono_ is the energy of the optimized isolated molecule.

### Thermal Analysis

Thermal behaviors of *p*Tol‐BTBT‐C*_n_* were analyzed by differential scanning calorimetry (DSC; DSC 8500, PerkinElmer Co., Ltd.) at a scanning rate of 5 K min^−1^ using powdered samples. The measured temperature was calibrated using the melting point of indium (429.8 K). The melting points of *p*Tol‐BTBT‐C*_n_* were also checked by visual observation of the changes in powdered samples on a hot plate. Figures S9 and S10 (Supporting Information), respectively, show the obtained DSC charts, and changes in enthalpy *ΔS* and entropy *ΔH*.

### Powder XRD Measurement

The powder XRD measurements were carried out at the beamline BL‐8B of Photon Factory (PF), High‐Energy Accelerator Research Organization (KEK). The used wavelength λ of the synchrotron X‐rays was 1.236 Å. A glass capillary with an inside diameter of ≈0.3 mm was filled with finely ground powder of the respective compounds, which was used for the diffraction measurement at 200 °C controlled by blowing of temperature‐regulated nitrogen gas.

### Thin Film Fabrication

Single‐crystal thin films of *p*Tol‐BTBT‐C*_n_* were all fabricated by the blade‐coating technique.^[^ ^34^, ^36^ ^]^ 0.05−0.1 wt.% semiconductor solution was used in chlorobenzene at sweep rates of 1.0−3.0 µm s^−1^ by a fluoropolymer‐coated glass blade at room temperature. The glass blade was coated with CYTOP by spin‐coating technique. Optical microscope images of the films were collected using a digital microscope (VHX‐6000; Keyence Co., Ltd.). The film thickness determined by height profiles of the films was measured by AFM (VN‐8010; Keyence Co., Ltd. and MFP‐3D; Asylum Research, USA).

### Thin Film XRD Measurements

Cover glasses (thickness; c.a. 200 µm) were used as substrates for determining crystal‐lattice parameters of the blade‐coated films, shown in Figure 5 and Table 1. Diffraction data were collected by the same procedure as single‐crystal XRD measurements. Synchrotron radiated X‐ray beam monochromatized was used at 1.55 Å at the beamline BL‐8A of the KEK Photon Factory. A few small‐angle Bragg diffractions were used shown in Table S5 (Supporting Information) for determining the crystal lattice. The measurement of out‐of‐plane and in‐plane XRD profiles was carried out with a thin‐film diffractometer (SOR‐SmartLab; Rigaku Co., Ltd.). Synchrotron radiated X‐ray beam monochromatized was used at 1.241 Å for *n* = 10 and at 1.377 Å for *n* = 11 at the beamline BL‐7C of the KEK Photon Factory. In the measurement, single‐crystal thin films fabricated were used on highly doped (*p*+)‐Si wafers with 100 nm‐thick silicon dioxide layers.

### Thin Film Optical SHG Measurements

Optical SHG measurements of single‐crystal thin‐films for *p*Tol‐BTBT‐C*_n_* onto glass substrate were performed with light pulses from a Ti:Sapphire laser with a central wavelength of 800 nm, pulse width of 100 fs, and repetition rate of 80 MHz. The light pulses were irradiated onto the single‐crystal thin‐film of *p*Tol‐BTBT‐C*_n_* at the setup exhibited in Figure 6C,D. The transmitted SHG lights were detected by a photomultiplier tube. The laser power and the focus diameter were ≈40 mW and ≈100 µm, respectively. A polarizer and a half‐wave plate were used to generate the polarized irradiation light, and an analyzer was used to detect the polarized SHG light. A long‐pass filter before the sample was used to block unwanted signals, and a short‐pass filter and a monochromator after the sample were used to separate the SHG light from the fundamental light.

### OFET Device Fabrication and Characterization

BGTC‐type single‐crystal OFETs were fabricated employing SiO_2_ or dix‐SR (parylene SR) as a gate insulator, respectively. In the fabrication of SiO_2_‐based OFETs, highly doped (*p*+)‐silicon wafers with 100 nm‐thick silicon dioxide layers (Si/SiO_2_ wafer) were used. In the fabrication of parylene‐based OFETs, A dix‐SR layer was deposited by chemical vapor deposition on the Si/SiO_2_ (100 nm) wafer. The thickness of the dix‐SR layer was evaluated to be 58 nm, and the resulting total capacitance was estimated to be 20.6 nF cm^−1^.^[^ ^44^ ^]^ The semiconductive layer was manufactured by the blade‐coating technique. Then the source/drain electrodes of Au were deposited in vacuum through a shadow mask. The channel length (L) and width (W) were defined as 200 and 300−500 µm, respectively, for all the devices. A micromanipulator (Axispro; Systems Engineering Inc.) was used to trim away the films outside the channels for the proper evaluation of the device mobility.

The OFET characteristics under ambient conditions were measured using a Source/Measure Unit (B2912A; Keysight Technologies Inc.). The field‐effect mobility (*µ*) in a saturation regime was calculated by using the following equation:

(2) $$\mu =\frac{2L}{WC_{i}}\;{\left(\frac{\partial \sqrt{I_{D}}}{\partial V_{G}}\right)}^{2}$$

where *I*_D_, *C*_i_, *V*_D_, and *V*_G_ are the drain current, gate capacitance per unit area, drain voltage, and gate voltage, respectively. After the measurements, out‐of‐plane XRD measurements were conducted for SiO_2_‐based devices to ensure the molecular arrangement of semiconductive layers, as depicted in Figure S20 (Supporting Information). The XRD measurements were carried out using synchrotron radiated X‐ray beam (λ = 1.377 Å) and a diffractometer (SOR‐Smartlab, Rigaku Co., Ltd.) installed at the beamline BL‐7C at Photon Factory, KEK.

## Conflict of Interest

The authors declare no conflict of interest.

## Supporting information

Supporting Information

## Data Availability

The data that support the findings of this study are available from the corresponding author upon reasonable request.
