# Supplementary material for: Control of Polar/Antipolar Layered Organic Semiconductors by the Odd‐Even Effect of Alkyl Chain
Source: Adv Sci (Weinh). 2024 Jan 25;11(13):2308270. doi: 10.1002/advs.202308270 (PMC10987142; doi:10.1002/advs.202308270)
Supplement: Supplementary file 1 — Supporting Information [file ADVS-11-2308270-s001.pdf]

ADVANCED SCIENCE
Open Access

Supporting Information

for *Adv. Sci.*, DOI 10.1002/advs.202308270

Control of Polar/Antipolar Layered Organic Semiconductors by the Odd-Even Effect of Alkyl Chain

Satoru Inoue, Toshiki Higashino, Kiyoshi Nikaido, Ryo Miyata, Satoshi Matsuoka, Mutsuo Tanaka, Seiji Tsuzuki, Sachio Horiuchi, Ryusuke Kondo, Ryoko Sagayama, Reiji Kumai, Daiki Sekine, Takayoshi Koyanagi, Masakazu Matsubara and Tatsuo Hasegawa**

Supporting Information

Control of Polar/Antipolar Layered Organic Semiconductors by the Odd-Even Effect of Alkyl Chain

Satoru Inoue*, Toshiki Higashino, Kiyoshi Nikaido, Ryo Miyata, Satoshi Matsuoka, Mutsuo Tanaka, Seiji Tsuzuki, Sachio Horiuchi, Ryusuke Kondo, Ryoko Sagayama, Reiji Kumai, Daiki Sekine, Takayoshi Koyanagi, Masakazu Matsubara, and Tatsuo Hasegawa*

*Corresponding author: satoru.inoue@ap.t.u-tokyo.ac.jp ; t-hasegawa@ap.t.u-tokyo.ac.jp

Materials Synthesis

All chemicals and solvents are of reagent grade unless otherwise indicated. Nuclear magnetic resonance (NMR) spectra were recorded on a JEOL JNM-ECS400 spectrometer at 400MHz. Chemical shifts (δ) are reported in ppm relative to tetramethylsilane (δ 0.00). The data of multiplicity are shown as follows: s = singlet, d = doublet, t = triplet, q = qualtet, m = multiplet. Mass analyses (MS) were performed on a Shimadzu GC-MS QP2010SE in an electron impact ionization procedure. Elemental analyses were collected on a J-Science Lab JM10. As shown in Figure S1, *p*Tol-BTBT-C_n were all synthesized using Suzuki coupling reaction between Br-BTBT-C_n and *para*-tolylboronic acid. Corresponding Br-BTBT-C_n were synthesized by the reported procedure[19].

General procedure for the synthesis of *p*Tol-BTBT-C_n: A mixture of corresponding Br-BTBT-C_n (1 mmol), *p*-tolylboronic acid (272 mg, 2 mmol, 2.0 eq), K_2CO_3 (276 mg, 5.0 eq), and $Pd(PPh_3)_4$ (57.8 mg, 5 mol%) in a mixed solution of toluene (50 mL) and H_2O (10 mL) was stirred for 5 h at 90 ºC. The organic layer was separated and passed through a silica gel pad with hexane-chloroform (5:1 v/v) as eluent. Purification of the white powdered product obtained by evaporation was finally carried out by recrystallization from a mixed solution of chloroform and ethanol to afford the corresponding *p*Tol-BTBT-C_n as white crystals.

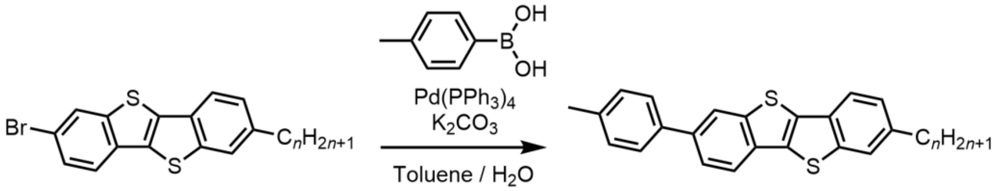

Figure S1. Synthetic scheme of *p*Tol-BTBT-C_n.

***p*Tol-BTBT-C_5**: 87 % yield. ^{1}H NMR (400 MHz, $CDCl_3$): δ (ppm) 8.10 (s, 1H, ArH), 7.90 (d, *J* = 8.0 Hz, 1H, ArH), 7.79 (d, *J* = 8.0 Hz, 1H, ArH), 7.73 (s, 1H, ArH), 7.68 (dd, *J* = 8.0, 2.0 Hz, 1H, ArH), 7.59 (d, *J* = 8.0 Hz, 2H, ArH), 7.29 (m, 3H, ArH), 2.77 (t, *J* = 8.0 Hz, 2H, CH_2), 2.42 (s, 3H, $ArCH_3$), 1.71 (tt, *J* = 8.0, 7.2 Hz, 2H, CH_2), 1.37 (m, 4H, CH_2), 0.91 (t, *J* = 7.2 Hz, 3H, CH_3). ^{13}C NMR (100MHz, $CDCl_3$); δ (ppm) 142.90, 142.64, 140.37, 138.05, 137.89, 137.25, 133.53, 132.40, 132.14, 131.09, 129.64, 127.13, 125.93, 124.28, 123.37, 122.04, 121.53, 121.22, 36.11, 31.50, 31.39, 22.57, 21.13, 14.04. MS (EI mode) m/z 400. Anal. Calcd for $C_{26}H_{24}S_2$: C, 77.95%, H, 6.04%. Found: C, 78.03%, H, 6.29%.

***p*Tol-BTBT-C$_6$**: 82 % yield. ^{1}H NMR (400 MHz, $CDCl_3$): δ (ppm) 8.10 (s, 1H, ArH), 7.90 (d, J = 8.0 Hz, 1H, ArH), 7.79 (d, J = 8.0 Hz, 1H, ArH), 7.73 (s, 1H, ArH), 7.68 (dd, J = 8.0, 2.0 Hz, 1H, ArH), 7.59 (d, J = 8.0 Hz, 2H, ArH), 7.29 (m, 3H, ArH), 2.77 (t, J = 8.0 Hz, 2H, CH_2), 2.42 (s, 3H, $ArCH_3$), 1.71 (tt, J = 8.0, 7.2 Hz, 2H, CH_2), 1.33 (m, 6H, CH_2), 0.90 (t, J = 7.2 Hz, 3H, CH_3). ^{13}C NMR (100MHz, $CDCl_3$); δ (ppm) 142.90, 142.64, 140.39, 138.06, 137.90, 137.26, 133.53, 132.41, 132.15, 131.10, 129.65, 127.14, 125.94, 124.30, 123.37, 122.05, 121.54, 121.22, 36.16, 31.75, 31.68, 29.00, 22.63, 21.14, 14.11. MS (EI mode) m/z 414. Anal. Calcd for $C_{27}H_{26}S_2$: C, 78.21%, H, 6.32%. Found: C, 78.20%, H, 6.53%.

***p*Tol-BTBT-C$_7$**: 84 % yield. ^{1}H NMR (400 MHz, $CDCl_3$): δ (ppm) 8.10 (s, 1H, ArH), 7.90 (d, J = 8.0 Hz, 1H, ArH), 7.79 (d, J = 8.0 Hz, 1H, ArH), 7.73 (s, 1H, ArH), 7.68 (dd, J = 8.0, 2.0 Hz, 1H, ArH), 7.59 (d, J = 8.0 Hz, 2H, ArH), 7.29 (m, 3H, ArH), 2.77 (t, J = 8.0 Hz, 2H, CH_2), 2.42 (s, 3H, $ArCH_3$), 1.71 (tt, J = 8.0, 7.2 Hz, 2H, CH_2), 1.35-1.27 (m, 8H, CH_2), 0.89 (t, J = 7.2 Hz, 3H, CH_3). ^{13}C NMR (100MHz, $CDCl_3$); δ (ppm) 142.90, 142.64, 140.39, 138.06, 137.90, 137.26, 133.53, 132.41, 132.15, 131.10, 129.65, 127.14, 125.94, 124.30, 123.37, 122.05, 121.54, 121.22, 36.16, 31.83, 31.72, 29.29, 29.20, 22.68, 21.14, 14.11. MS (EI mode) m/z 428.2. Anal. Calcd for $C_{28}H_{28}S_2$: C, 78.46%, H, 6.58%. Found: C, 78.61%, H, 6.80%.

***p*Tol-BTBT-C$_8$**: 75 % yield. ^{1}H NMR (400 MHz, $CDCl_3$): δ (ppm) 8.10 (s, 1H, ArH), 7.90 (d, J = 8.0 Hz, 1H, ArH), 7.79 (d, J = 8.0 Hz, 1H, ArH), 7.73 (s, 1H, ArH), 7.68 (dd, J = 8.0, 2.0 Hz, 1H, ArH), 7.59 (d, J = 8.0 Hz, 2H, ArH), 7.29 (m, 3H, ArH), 2.77 (t, J = 8.0 Hz, 2H, CH_2), 2.42 (s, 3H, $ArCH_3$), 1.70 (tt, J = 8.0, 7.2 Hz, 2H, CH_2), 1.35-1.27 (br, 10H, CH_2), 0.88 (t, J = 7.2 Hz, 3H, CH_3). ^{13}C NMR (100MHz, $CDCl_3$); δ (ppm) 142.90, 142.64, 140.39, 138.06, 137.90, 137.26, 133.53, 132.41, 132.15, 131.10, 129.65, 127.14, 125.94, 124.30, 123.37, 122.05, 121.54, 121.22, 36.16, 31.89, 31.72, 29.50, 29.34, 29.27, 22.68, 21.14, 14.12. MS (EI mode) m/z 442. Anal. Calcd for $C_{29}H_{30}S_2$: C, 78.68%, H, 6.83%. Found: C, 78.73%, H, 7.02%.

***p*Tol-BTBT-C$_9$**: 77 % yield. ^{1}H NMR (400 MHz, $CDCl_3$): δ (ppm) 8.10 (s, 1H, ArH), 7.90 (d, J = 8.0 Hz, 1H, ArH), 7.79 (d, J = 8.0 Hz, 1H, ArH), 7.73 (s, 1H, ArH), 7.68 (dd, J = 8.0, 2.0 Hz, 1H, ArH), 7.59 (d, J = 8.0 Hz, 2H, ArH), 7.29 (m, 3H, ArH), 2.77 (t, J = 8.0 Hz, 2H, CH_2), 2.42 (s, 3H, $ArCH_3$), 1.70 (tt, J = 8.0, 7.2 Hz, 2H, CH_2), 1.35-1.27 (br, 12H, CH_2), 0.88 (t, J = 7.2 Hz, 3H, CH_3). ^{13}C NMR (100MHz, $CDCl_3$); δ (ppm) 142.90, 142.64, 140.39, 138.06, 137.90, 137.26, 133.53, 132.41, 132.15, 131.10, 129.65, 127.14, 125.94, 124.30, 123.37, 122.05, 121.54, 121.22, 36.17, 31.92, 31.74, 29.59, 29.57, 29.36, 29.36, 22.70, 21.16,

14.14. MS (EI mode) m/z 456. Anal. Calcd for $C_{30}H_{32}S_2$: C, 78.90%, H, 7.06%. Found: C, 78.90%, H, 7.16%.

***p*Tol-BTBT-C$_{10}$**: 64 % yield. ^{1}H NMR (400 MHz, $CDCl_3$): δ (ppm) 8.10 (s, 1H, ArH), 7.90 (d, *J* = 8.0 Hz, 1H, ArH), 7.79 (d, *J* = 8.0 Hz, 1H, ArH), 7.73 (s, 1H, ArH), 7.68 (dd, *J* = 8.0, 2.0 Hz, 1H, ArH), 7.59 (d, *J* = 8.0 Hz, 2H, ArH), 7.29 (m, 3H, ArH), 2.77 (t, *J* = 8.0 Hz, 2H, CH_2), 2.42 (s, 3H, $ArCH_3$), 1.70 (tt, *J* = 8.0, 7.2 Hz, 2H, CH_2), 1.35-1.27 (br, 14H, CH_2), 0.88 (t, *J* = 7.2 Hz, 3H, CH_3). ^{13}C NMR (100MHz, $CDCl_3$); δ (ppm) 142.90, 142.64, 140.39, 138.06, 137.90, 137.26, 133.53, 132.41, 132.15, 131.10, 129.65, 127.14, 125.94, 124.30, 123.37, 122.05, 121.54, 121.22, 36.16, 31.91, 31.71, 29.62, 29.61, 29.53, 29.34(2C), 22.69, 21.14, 14.12. MS (EI mode) m/z 470. Anal. Calcd for $C_{31}H_{34}S_2$: C, 79.10%, H, 7.28%. Found: C, 79.03%, H, 7.30%.

***p*Tol-BTBT-C$_{11}$**: 72 % yield. ^{1}H NMR (400 MHz, $CDCl_3$): δ (ppm) 8.10 (s, 1H, ArH), 7.90 (d, *J* = 8.0 Hz, 1H, ArH), 7.79 (d, *J* = 8.0 Hz, 1H, ArH), 7.73 (s, 1H, ArH), 7.68 (dd, *J* = 8.0, 2.0 Hz, 1H, ArH), 7.59 (d, *J* = 8.0 Hz, 2H, ArH), 7.29 (m, 3H, ArH), 2.77 (t, *J* = 8.0 Hz, 2H, CH_2), 2.42 (s, 3H, $ArCH_3$), 1.70 (tt, *J* = 8.0, 7.2 Hz, 2H, CH_2), 1.35-1.27 (br, 16H, CH_2), 0.88 (t, *J* = 7.2 Hz, 3H, CH_3). ^{13}C NMR (100MHz, $CDCl_3$); δ (ppm) 142.90, 142.64, 140.39, 138.06, 137.90, 137.26, 133.53, 132.41, 132.15, 131.10, 129.65, 127.14, 125.94, 124.30, 123.37, 122.05, 121.54, 121.22, 36.16, 31.92, 31.71, 29.67, 29.63, 29.60, 29.54, 29.35, 29.32, 22.69, 21.14, 14.13. MS (EI mode) m/z 484. Anal. Calcd for $C_{32}H_{36}S_2$: C, 79.29%, H, 7.49%. Found: C, 79.16%, H, 7.51%.

***p*Tol-BTBT-C$_{12}$**: 92 % yield. ^{1}H NMR (400 MHz, $CDCl_3$): δ (ppm) 8.10 (s, 1H, ArH), 7.90 (d, *J* = 8.0 Hz, 1H, ArH), 7.79 (d, *J* = 8.0 Hz, 1H, ArH), 7.73 (s, 1H, ArH), 7.68 (dd, *J* = 8.0, 2.0 Hz, 1H, ArH), 7.59 (d, *J* = 8.0 Hz, 2H, ArH), 7.29 (m, 3H, ArH), 2.77 (t, *J* = 8.0 Hz, 2H, CH_2), 2.42 (s, 3H, $ArCH_3$), 1.70 (tt, *J* = 8.0, 7.2 Hz, 2H, CH_2), 1.35-1.27 (br, 18H, CH_2), 0.88 (t, *J* = 7.2 Hz, 3H, CH_3). ^{13}C NMR (100MHz, $CDCl_3$); δ (ppm) 142.90, 142.64, 140.39, 138.06, 137.90, 137.26, 133.53, 132.41, 132.15, 131.10, 129.65, 127.14, 125.94, 124.30, 123.37, 122.05, 121.54, 121.22, 36.15, 31.93, 31.71, 29.67(3C), 29.60, 29.53, 29.36, 29.31, 22.70, 21.14, 14.13. MS (EI mode) m/z 498. Anal. Calcd for $C_{33}H_{38}S_2$: C, 79.47%, H, 7.68%. Found: C, 79.09%, H, 7.63%.

***p*Tol-BTBT-C$_{13}$**: 59 % yield. ^{1}H NMR (400 MHz, $CDCl_3$): δ (ppm) 8.10 (s, 1H, ArH), 7.90 (d, *J* = 8.0 Hz, 1H, ArH), 7.79 (d, *J* = 8.0 Hz, 1H, ArH), 7.73 (s, 1H, ArH), 7.68 (dd, *J* = 8.0, 2.0 Hz, 1H, ArH), 7.59 (d, *J* = 8.0 Hz, 2H, ArH), 7.29 (m, 3H, ArH), 2.77 (t, *J* = 8.0 Hz, 2H, CH_2), 2.42 (s, 3H, $ArCH_3$), 1.70 (tt, *J* = 8.0, 7.2 Hz, 2H, CH_2), 1.35-1.27 (br, 20H, CH_2), 0.88

(t, J = 7.2 Hz, 3H, CH_3). ^{13}C NMR (100MHz, $CDCl_3$); δ (ppm) 142.90, 142.64, 140.39, 138.06, 137.90, 137.26, 133.53, 132.41, 132.15, 131.10, 129.65, 127.14, 125.94, 124.30, 123.37, 122.05, 121.54, 121.22, 36.15, 31.93, 31.71, 29.67(4C), 29.60, 29.53, 29.36, 29.31, 22.70, 21.14, 14.13. MS (EI mode) m/z 512. Anal. Calcd for $C_{34}H_{40}S_2$: C, 79.63%, H, 7.86%. Found: C, 79.53%, H, 7.80%.

***p*Tol-BTBT-C_{14}**: 61 % yield. ^1H NMR (400 MHz, $CDCl_3$): δ (ppm) 8.10 (s, 1H, ArH), 7.90 (d, J = 8.0 Hz, 1H, ArH), 7.79 (d, J = 8.0 Hz, 1H, ArH), 7.73 (s, 1H, ArH), 7.68 (dd, J = 8.0, 2.0 Hz, 1H, ArH), 7.59 (d, J = 8.0 Hz, 2H, ArH), 7.29 (m, 3H, ArH), 2.77 (t, J = 8.0 Hz, 2H, CH_2), 2.42 (s, 3H, $ArCH_3$), 1.70 (tt, J = 8.0, 7.2 Hz, 2H, CH_2), 1.35-1.27 (br, 22H, CH_2), 0.88 (t, J = 7.2 Hz, 3H, CH_3). ^{13}C NMR (100MHz, $CDCl_3$); δ (ppm) 142.90, 142.64, 140.39, 138.06, 137.90, 137.26, 133.53, 132.41, 132.15, 131.10, 129.65, 127.14, 125.94, 124.30, 123.37, 122.05, 121.54, 121.22, 36.15, 31.93, 31.71, 29.67(5C), 29.60, 29.53, 29.37, 29.31, 22.70, 21.11, 14.13. MS (EI mode) m/z 526. Anal. Calcd for $C_{35}H_{42}S_2$: C, 79.79%, H, 8.04%. Found: C, 79.35%, H, 7.75%.

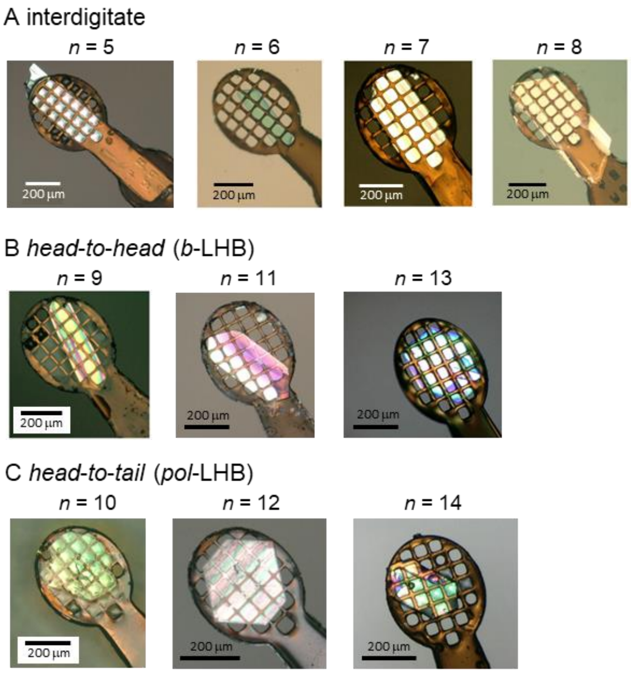

Figure S2. Optical micrographs for single crystals of *p*Tol-BTBT-C$_n$ for crystal structure analyses. Single crystals for (A) n = 5−8, (B) n = 9, 11, and 13, (C) n = 10, 12, and 14.

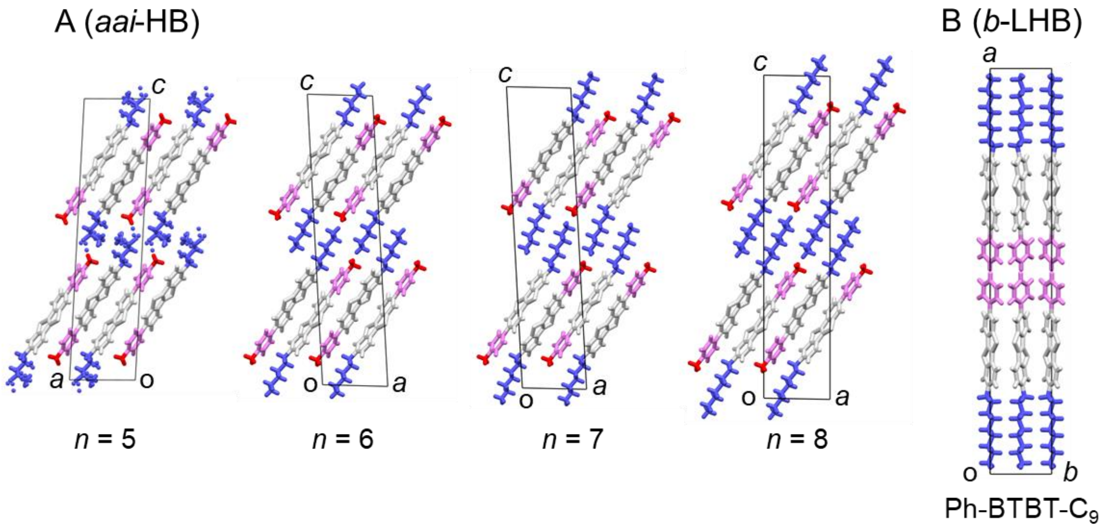

Figure S3. Packing diagrams of *p*Tol-BTBT-C$_n$ (n = 5–8) and Ph-BTBT-C$_9$. (A) n = 5–8 in *p*Tol-BTBT-C$_n$ projected along the *a*-*c* plane. (B) Ph-BTBT-C$_9$ projected along the *a*-*b* plane. The BTBT moiety are shown by the color of white, whereas alkyl chains, phenyl groups, and end methyl groups by blue, pink, and red.

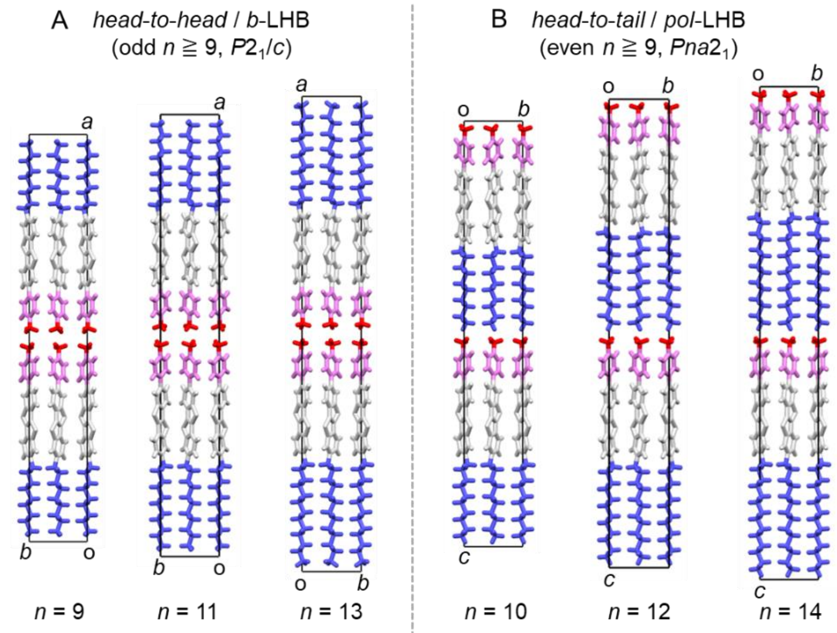

Figure S4. Packing diagrams of *p*Tol-BTBT-C$_n$ (n = 9−14). (A) n = 9, 11, and 13 projected along the *a*-*b* plane. (B) n = 10, 12, and 14 projected along the the *b*-*c* plane. The BTBT moiety are shown by the color of white, whereas alkyl chains, phenyl groups, and end methyl groups by blue, pink, and red.

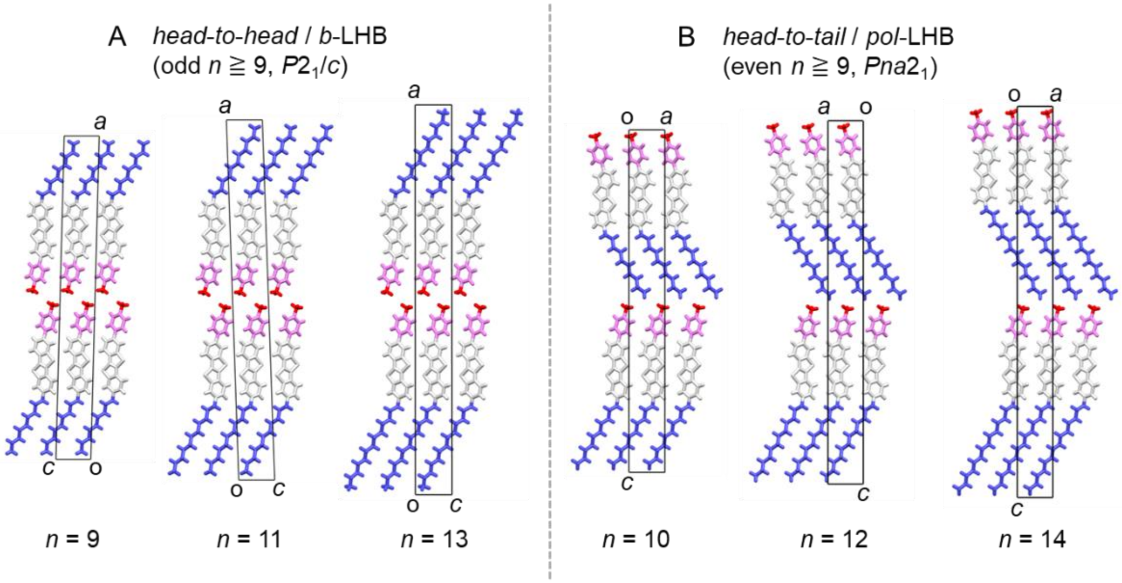

Figure S5. Packing diagrams of *p*Tol-BTBT-C$_n$ (n = 9−14). (A) n = 9, 11, and 13 projected along the *a*-*c* plane. (B) n = 10, 12, and 14 projected along the *a*-*c* plane. The BTBT moiety are shown by the color of white, whereas alkyl chains, phenyl groups, and end methyl groups by blue, pink, and red.

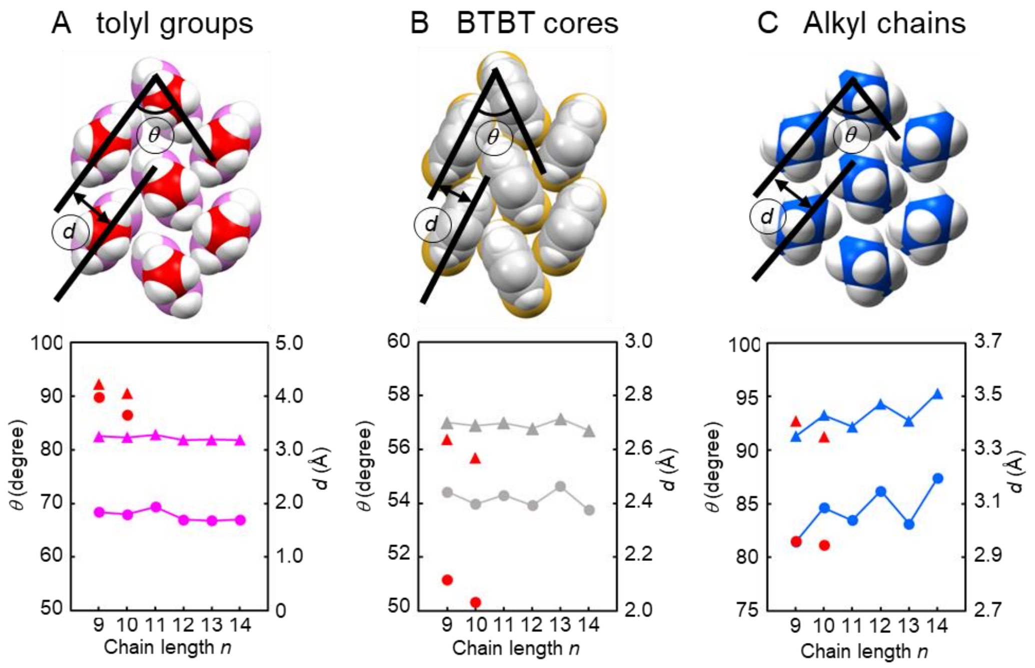

Figure S6. Variation of intralayer molecular packing arrangement in *p*Tol-BTBT-C_n (n = 9−14). (A) Tolyl groups. (B) BTBT cores. (C) Alkyl chains. The changes in dihedral angles θ (○) and mean interplanar distance d (△) are plotted as a function of alkyl chain length n. Definition of the dihedral angles and mean interplanar distances are shown in the space-filling views. Red plots show the results of Ph-BTBT-C_n (n = 9 and 10).

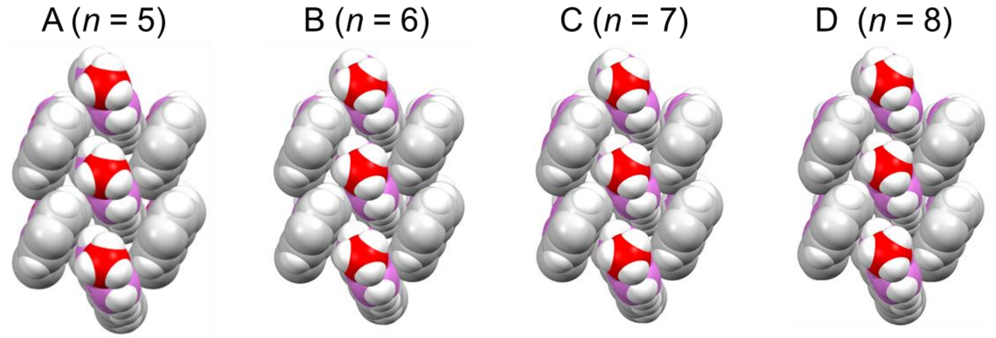

Figure S7. Intralayer molecular packing arrangements of BTBT cores and tolyl groups in *p*Tol-BTBT-C_n (n = 5−8). (A) n = 5. (B) n = 6. (C) n = 7. (D) n = 8.

Table S1. Crystallographic parameters of Ph-BTBT-C_9 and *p*Tol-BTBT-C_n.

Compounds	Ph-BTBT-C_9	*p*Tol-BTBT-C_5	*p*Tol-BTBT-C_6	*p*Tol-BTBT-C_7	*p*Tol-BTBT-C_8
Chemical formula	$C_{29}H_{30}S_2$	$C_{26}H_{24}S_2$	$C_{27}H_{26}S_2$	$C_{28}H_{28}S_2$	$C_{29}H_{30}S_2$
Formula weight	442.68	400.60	414.62	428.65	442.68
Crystal system	monoclinic	monoclinic	monoclinic	monoclinic	monoclinic
Space group	$P2_1/c$	$P2_1/c$	$P2_1/n$	$P2_1/n$	$P2_1/n$
a (Å)	51.657(9)	9.1167(5)	9.1363(5)	9.1572(3)	9.0903(4)
b (Å)	7.8515(14)	5.9715(3)	5.9272(2)	5.9434(2)	5.9152(3)
c (Å)	6.1099(9)	39.007(2)	40.763(2)	43.0458(15)	44.417(2)
α (deg)	90	90	90	90	90
β (deg)	90.843(15)	92.845(5)	94.484(5)	94.237(3)	91.059(4)
γ (deg)	90	90	90	90	90
V (Å^3)	2477.8(7)	2120.94(19)	2200.67(18)	2336.36(14)	2387.94(19)
Z vaule	4	4	4	4	4
Dcalc (g/cm^3)	1.187	1.254	1.251	1.219	1.231
Temperature	300	300	300	300	300
Radiation	MoKα	MoKα	MoKα	MoKα	MoKα
No. of reflections	5742	4864	5014	5264	5346
No. of valuables	280	253	262	271	280
R (I>2σ(I))	0.1558	0.0717	0.0763	0.0744	0.1421
wR^2	0.3614	0.1817	0.1290	0.1721	0.3691

Compounds	*p*Tol-BTBT-C_9	*p*Tol-BTBT-C_{10}	*p*Tol-BTBT-C_{11}	*p*Tol-BTBT-C_{12}	*p*Tol-BTBT-C_{13}	*p*Tol-BTBT-C_{14}
Chemical formula	$C_{30}H_{32}S_2$	$C_{31}H_{34}S_2$	$C_{32}H_{36}S_2$	$C_{33}H_{38}S_2$	$C_{34}H_{40}S_2$	$C_{35}H_{42}S_2$
Formula weight	456.70	470.73	484.78	498.78	512.78	526.84
Crystal system	monoclinic	orthorhombic	monoclinic	orthorhombic	monoclinic	orthorhombic
Space group	$P2_1/c$	$Pna2_1$	$P2_1/c$	$Pna2_1$	$P2_1/c$	$Pna2_1$
a (Å)	54.718(2)	5.92852(18)	59.180(6)	5.90933(13)	63.005(18)	5.9024(5)
b (Å)	7.8060(4)	7.7655(2)	7.8152(8)	7.7398(2)	7.852(3)	7.7178(8)
c (Å)	5.9079(3)	56.2232(16)	5.9147(5)	60.5371(17)	5.9208(16)	64.954(4)
α (deg)	90	90	90	90	90	90
β (deg)	91.482(4)	90	91.9725(16)	90	90.041(6)	90
γ (deg)	90	90	90	90	90	90
V (Å^3)	2522.6(2)	2588.40(13)	2733.9(5)	2768.79(12)	2929.0(15)	2958.9(4)
Z vaule	4	4	4	4	4	4
Dcalc (g/cm^3)	1.202	1.208	1.178	1.196	1.163	1.183
Temperature	300	300	300	300	300	300
Radiation	MoKα	MoKα	synchrotron (λ = 1.000 Å)	MoKα	synchrotron (λ = 1.233 Å)	MoKα
No. of reflections	5772	6583	1879	5425	2501	6673
No. of valuables	289	298	207	316	227	334
R (I>2σ(I))	0.1348	0.0622	0.0908	0.0434	0.1668	0.1016
wR^2	0.2932	0.1234	0.2226	0.0972	0.4660	0.1636

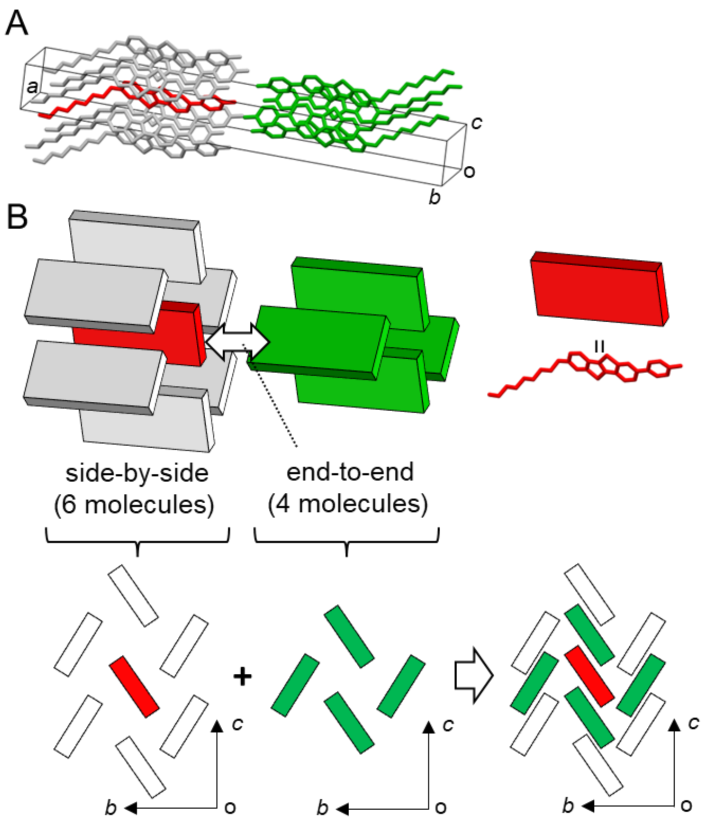

Figure S8. The *side-by-side* and *end-to-end* arrangements of *p*Tol-BTBT-C_n in LHB packing. (A) The packing diagram of *p*Tol-BTBT-C_n (n = 9−14) without hydrogen atoms. (B) Schematic images of the intermolecular arrangement. Respective plates represent the *p*Tol-BTBT-C_n molecule at the center (red), six neighboring *p*Tol-BTBT-C_n molecules at the *side-by-side* contacts within the same layer (gray), and four neighboring *p*Tol-BTBT-C_9 molecules at the *end-to-end* contacts in the adjacent layer.

Table S2. Intermolecular interaction energies[a)] in crystal packings of *p*Tol-BTBT-C_n and Ph-BTBT-C_n.

Materials	Chain length *n*	*Side-by-side* interaction energy (kcal/mol)	*End-to end* interaction energy (kcal/mol)		
			Alkyl-alkyl	Tolyl-tolyl (Ph-Ph)	Alkyl-tolyl
*p*Tol-BTBT-C_n	9	−93.3	−2.73	−3.18	–
	10	−97.7	–	–	−3.80
	11	−100.4	−2.66	−3.21	-
	12	−104.7	–	–	−3.89
	13	−105.8	−2.94	−3.17	–
	14	−111.5	–	–	−3.89
Ph-BTBT-C_n	9	−87.4	−2.72	−6.68	
	10	−93.1	−3.57	−6.67	

a) Calculated using Gaussian 16 program[50] at the PBE/6-311G** level with Grimme's D3BJ dispersion correction[51]. The basis set superposition error (BSSE) [52] was corrected by the counterpoise method[53]. The sum of the intermolecular interaction energies of a molecule with surrounding molecules in the same layer (*side-by-side*) or with those in the next layer (*end-to-end*) is shown. The sum of the interaction energies with surrounding molecules with the shortest interatomic distance of less than 8 Å in the crystal is shown.

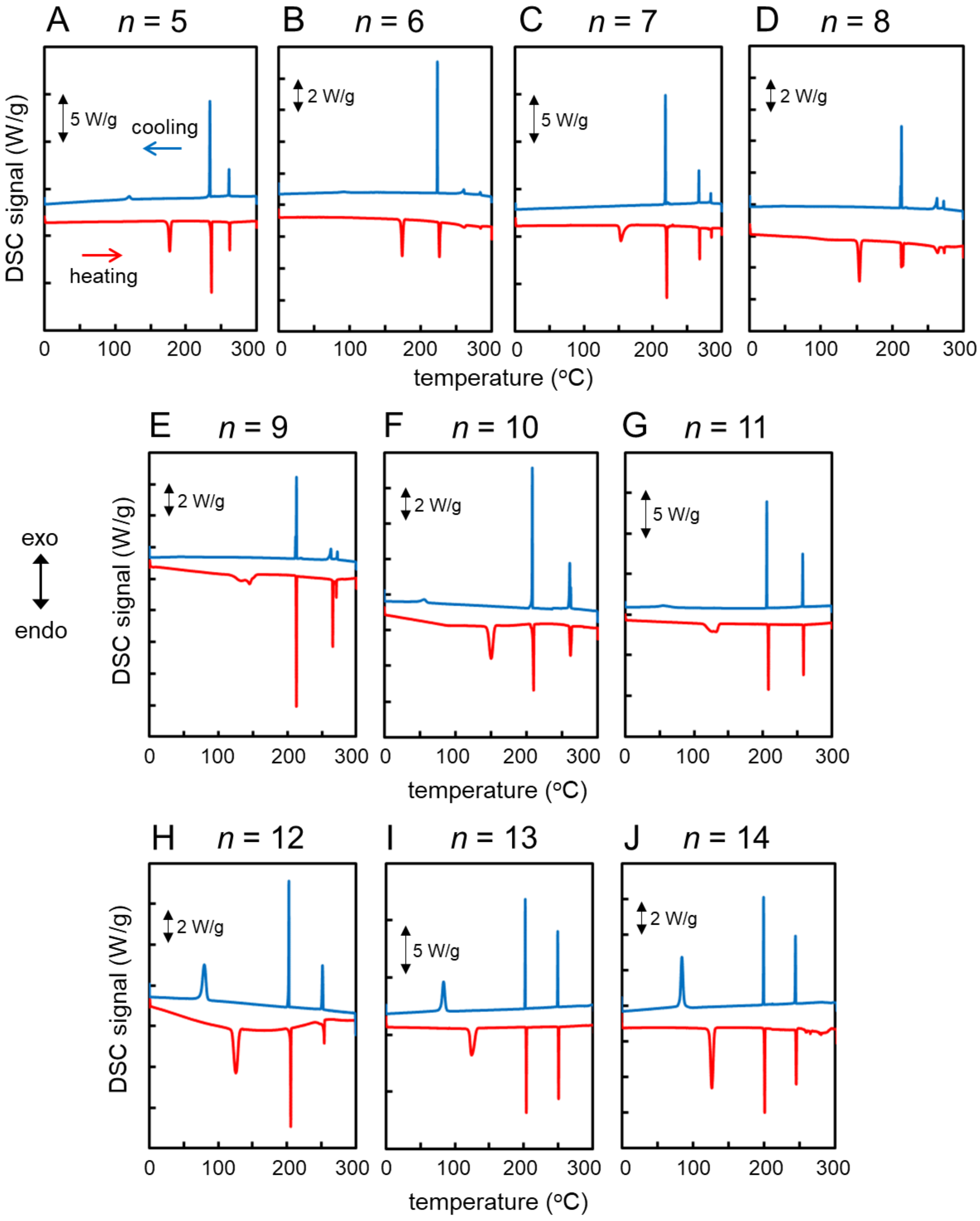

Figure S9. DSC charts of *p*Tol-BTBT-C$_n$ measured at 5 K/min. (A) n = 5. (B) n = 6. (C) n = 7. (D) n = 8. (E) n = 9. (F) n = 10. (G) n = 11. (H) n = 12. (I) n = 13. (J) n = 14. Red curves and blue curves show the results for first heating run and subsequent cooling run, respectively.

Table S3. List of phase transition temperature of *p*Tol-BTBT-C_n.

Chain length *n*	Phase transition temperature (1st heating, ºC)		
	Crystal ⇒ LC	LC ⇒ LC	Melting point
5	174.6	235.2	261.4
6	172.0	225.3, 254.2	282.5
7	150.5	219.2, 267.3	284.6
8	151.3	212.3, 259.0	272.5
9	119.2	212.5, 265.4	270.6
10	145.0	208.6	260.1
11	113.8	207.3	258.4
12	120.0	204.9	253.1
13	120.3	203.1	250.0
14	123.2	200.2	244.5

Table S4. List of solubility in chlorobenzene at room temperature.

Chain length *n*	Solubility (wt%)	
	*p*Tol-BTBT-C_n	Ph-BTBT-C_n[19]
5	0.96	0.90
6	0.69	0.67
7	0.86	0.71
8	0.55	0.42
9	0.98	0.47
10	0.35	0.22
11	0.46	0.25
12	0.28	0.136
13	0.26	0.140
14	0.12	0.098

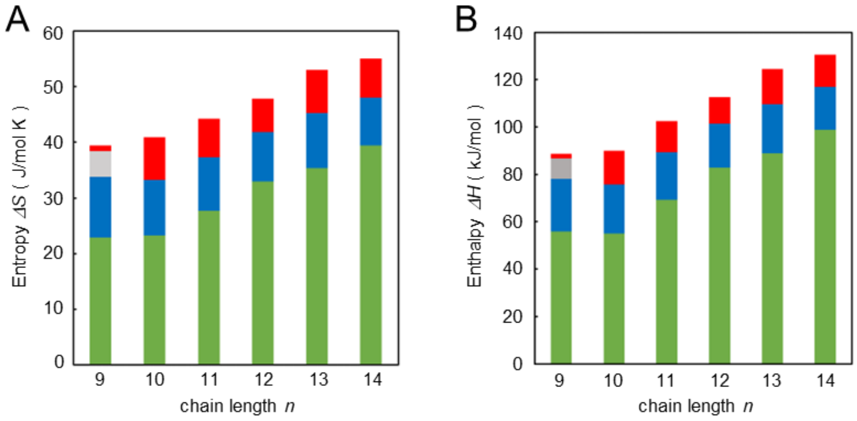

Figure S10. Changes in enthalpy ΔS and entropy ΔH of *p*Tol-BTBT-C$_n$ calculated from DSC charts in the heating step. (A) Changes in ΔS (=$\Delta H/T_{\mathrm{trans}}$, T_{trans}: phase transition temperature). (B) Changes in ΔH. Green: crystal to smectic E phase transition, blue: smectic E to liquid-crystal phase transition, red: melting point, gray: phase transition at 265 °C for n = 9 (where the peak was observed before melting point).

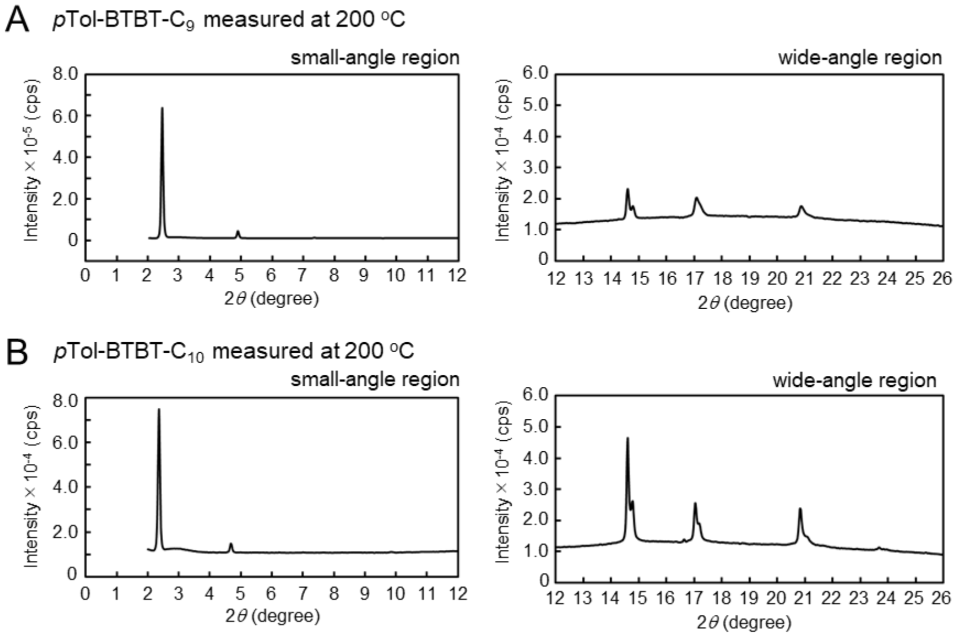

Figure S11. Powder XRD profiles of *p*Tol-BTBT-C$_n$ measured at 200 °C. (A) *p*Tol-BTBT-C$_9$. (B) *p*Tol-BTBT-C$_{10}$.

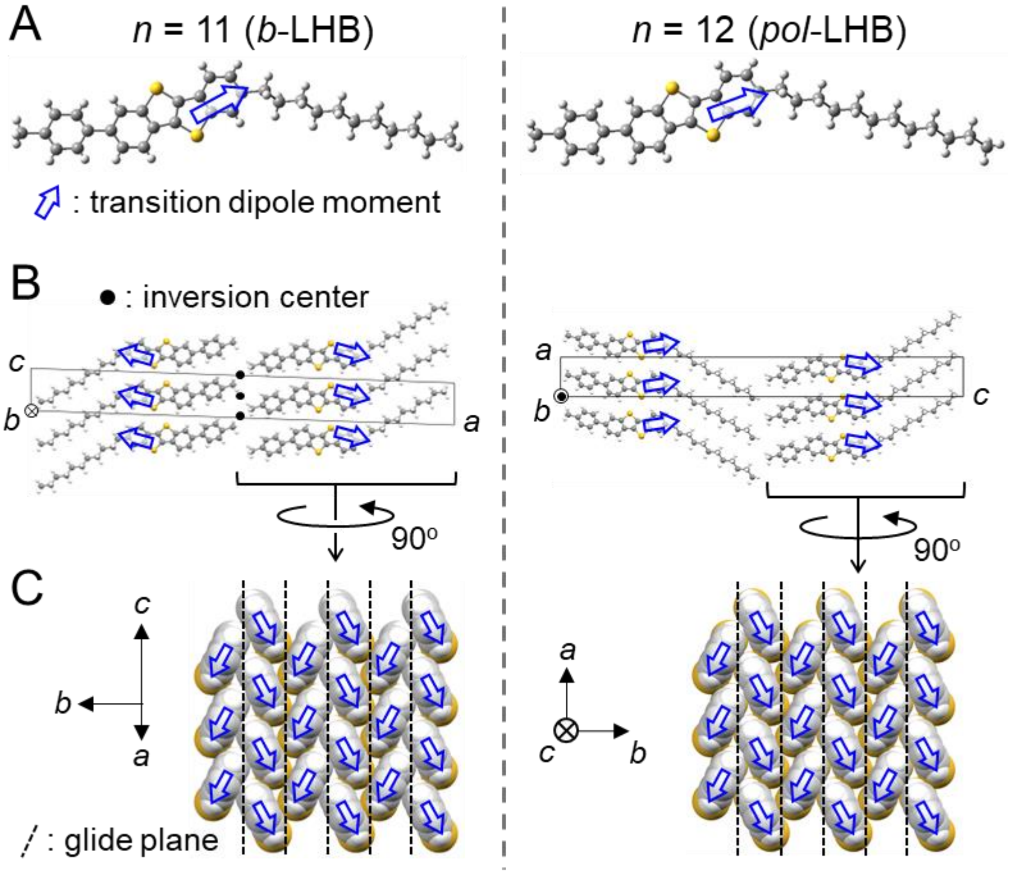

Figure S12. Transition dipole moments of *p*Tol-BTBT-C$_n$ crystals calculated by time-dependent DFT. (A) The direction of transition dipole moment (blue arrow) in *p*Tol-BTBT-C$_n$ molecules. (B) The direction of transition dipole moments in interlayer arrangements. (C) The direction of transition dipole moments within monomolecular layers.

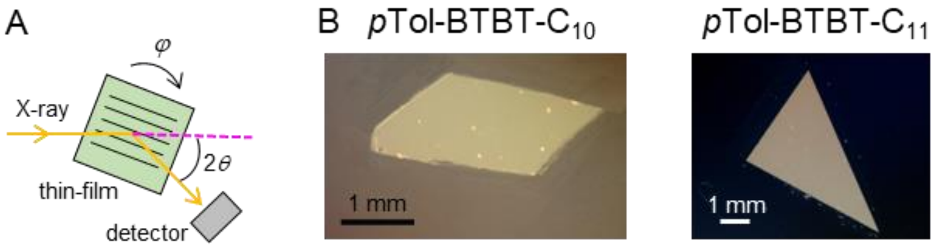

Figure S13. Experimental setup for in-plane XRD measurements of *p*Tol-BTBT-C$_n$ single crystal thin films. (A) Schematic experimental setup for in-plane XRD measurements of single-crystal films. (B) Crossed-Nicols polarized micrographs for isolated single crystal domain film of *p*Tol-BTBT-C$_{10}$ (left, thickness: 20 nm) and *p*Tol-BTBT-C$_{11}$ (right, thickness: 16 nm) used for the thin film XRD (out-of-plane and in-plane) measurements.

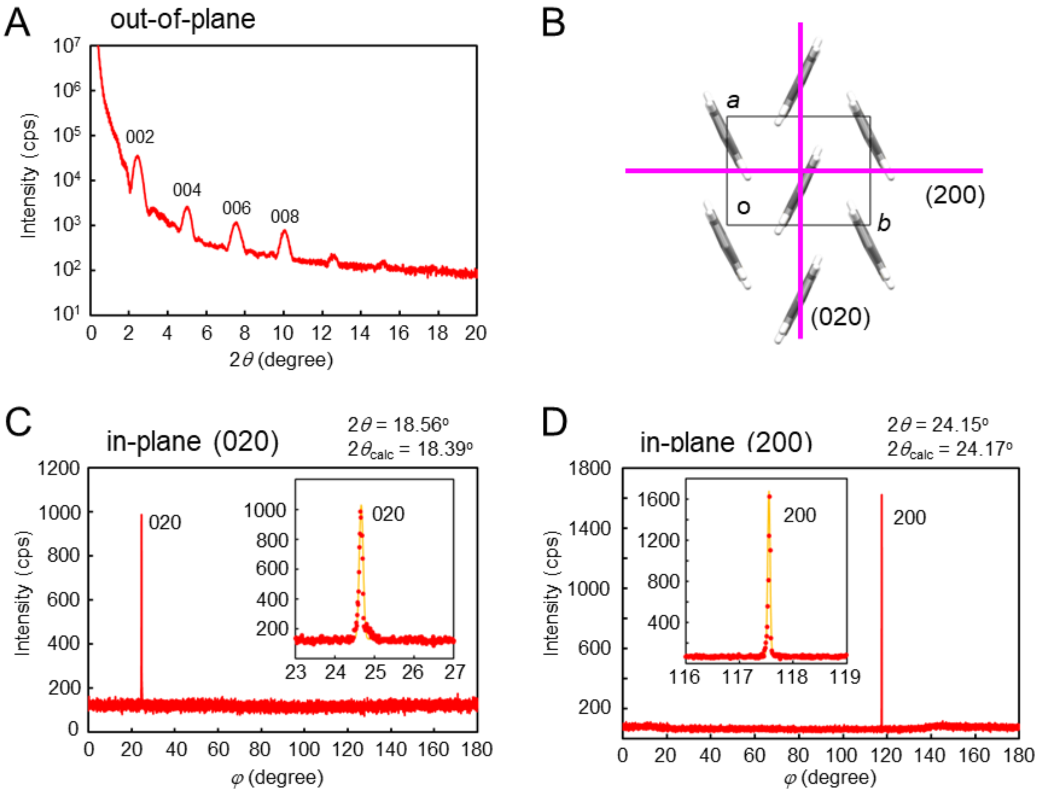

Figure S14. Thin film XRD measurement of the single-crystal thin film of *p*Tol-BTBT-C$_{10}$. (A) Out-of-plane XRD profiles. (B) Schematics for the arrangement of BTBT cores in *a*-*b* plane (in-plane) obtained by the crystallographic data of *p*Tol-BTBT-C$_{10}$ bulk crystal. Purple lines show (200) and (020) plane, respectively. (C)(D) In-plane-XRD profiles. $2\theta_{calc}$ means the value calculated from the crystallographic data of bulk crystal. The used wavelength λ of the synchrotron X-rays was = 1.241 Å.

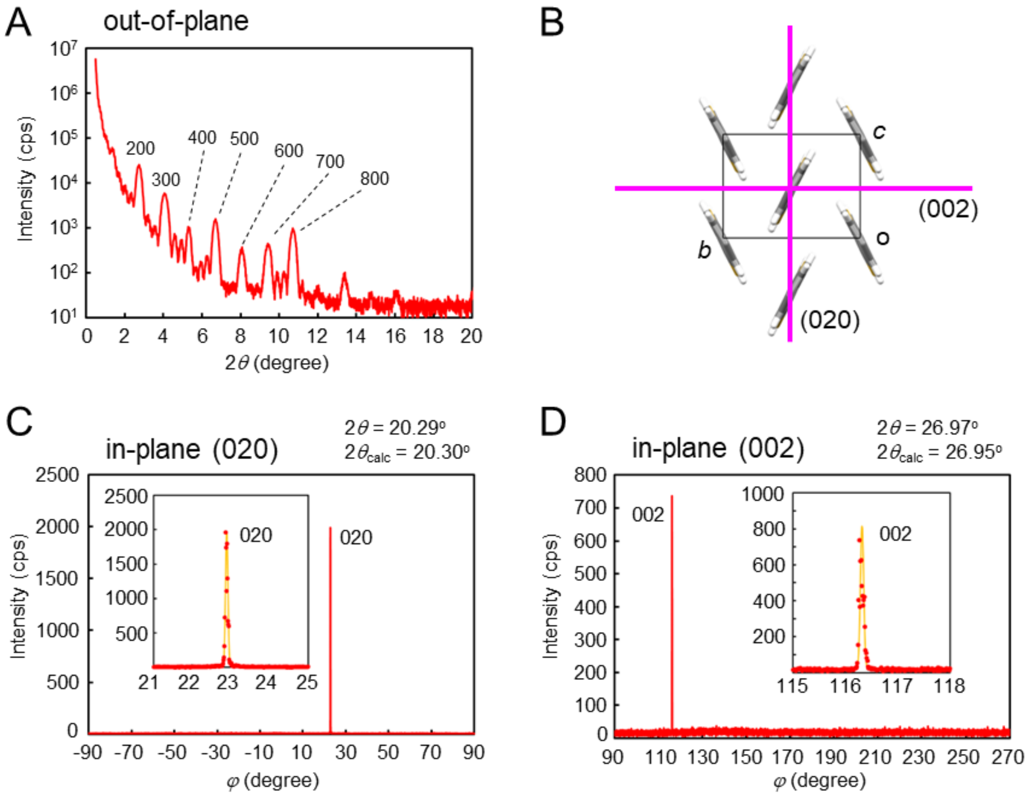

Figure S15. Thin film XRD measurement of the single-crystal thin film of *p*Tol-BTBT-C_{11}. (A) Out-of-plane XRD profiles. (B) Schematics for the arrangement of BTBT cores in *a*-*b* plane (in-plane) obtained by the crystallographic data of *p*Tol-BTBT-C_{11} bulk crystal. Purple lines show (200) and (020) plane, respectively. (C)(D) In-plane-XRD profiles. $2\theta_{calc}$ means the value calculated from the crystallographic data of bulk crystal. The used wavelength λ of the synchrotron X-rays was = 1.377 Å.

Table S5. Miller indices using the lattice constant of the single-crystal thin-film.

$n = 10$	$n = 11$
(1,1,1)	(0,1,1)
(1,$\bar{1}$,1)	(0,$\bar{1}$,1)
(1,1,$\bar{1}$)	(0,1,$\bar{1}$)
(1,$\bar{1}$,$\bar{1}$)	(0,$\bar{1}$,$\bar{1}$)
($\bar{1}$,1,1)	(3,1,$\bar{1}$)
($\bar{1}$,$\bar{1}$,1)	(3,$\bar{1}$,$\bar{1}$)
($\bar{1}$,1,$\bar{1}$)	($\bar{3}$,$\bar{1}$,1)
($\bar{1}$,$\bar{1}$,$\bar{1}$)	($\bar{3}$,1,1)

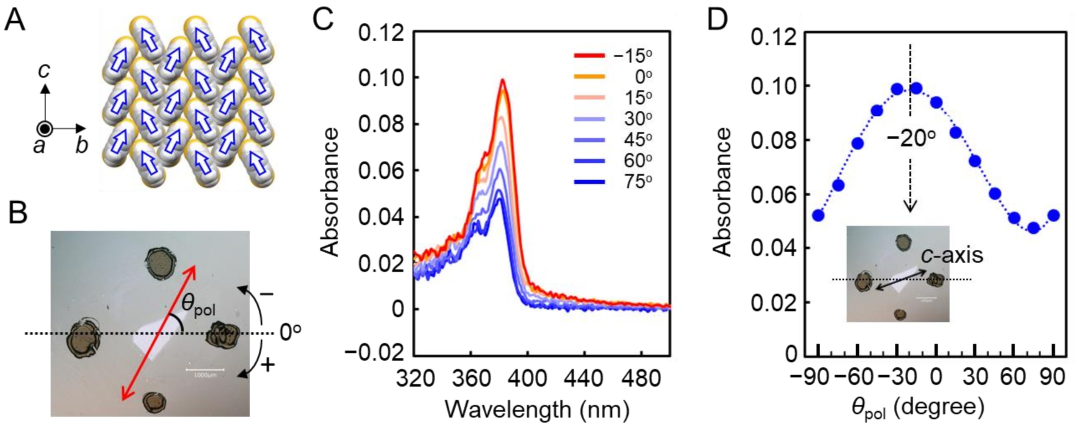

Figure S16. Determination of the crystallographic orientation in the single-crystal thin-film of *p*Tol-BTBT-C_{11}. (A) Arrangements of calculated transition dipole moment within the monomolecular layer. (B) Definition of the polarization angle (θ_{pol}). (C) Polarized absorption spectra. (D) Polarization angle dependence of the absorbance. The angle at which absorption becomes maximum aligns with the *c*-axis direction in the thin film. The *c*-axis of the single-crystal thin-film is oriented at the angle where absorption shows its maximum value.

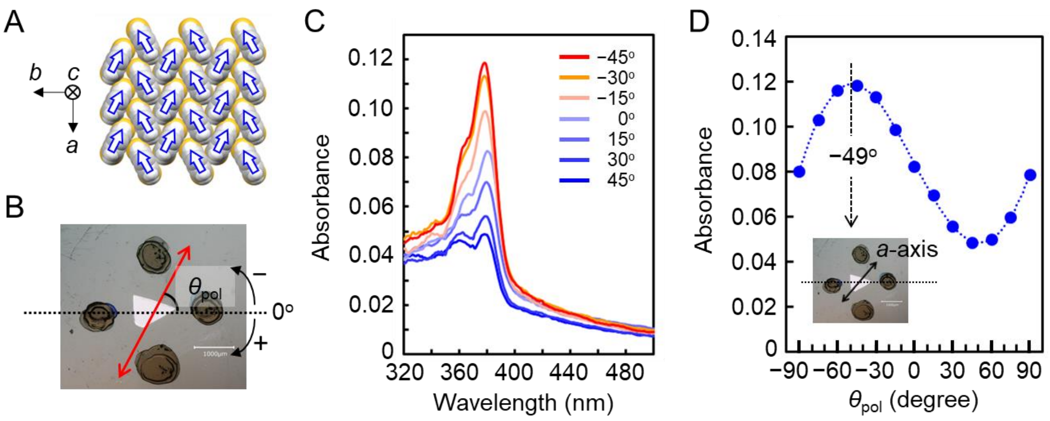

Figure S17. Determination of the crystallographic orientation in the single-crystal thin-film of *p*Tol-BTBT-C_{12}. (A) Arrangements of calculated transition dipole moment within the monomolecular layer. (B) Definition of the polarization angle (θ_{pol}). (C) The polarized absorption spectra. (D) Polarization angle dependence of the absorbance. The angle at which absorption becomes maximum aligns with the *a*-axis direction in the thin film. The *a*-axis of the single-crystal thin-film is oriented at the angle where absorption shows its maximum value.

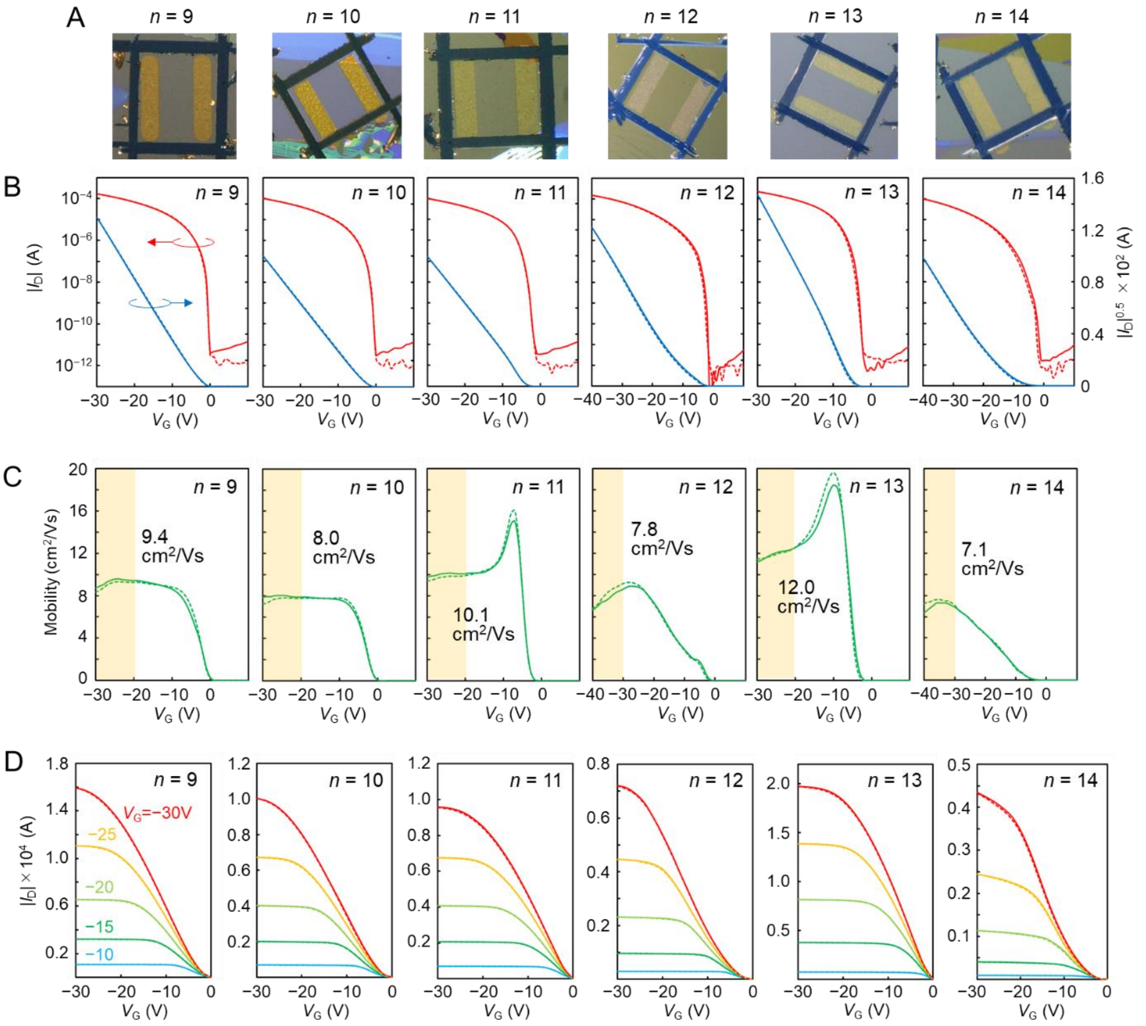

Figure S18. Sigle-crystal OFET device performance employing parylene gate insulator. (A) Optical microscope images of OFET devices for *p*Tol-BTBT-C_n. (B) Typical transfer characteristics for *p*Tol-BTBT-C_n single crystals measured at $V_D = -30$V (−40V for n = 12 and 14). Solid and dashed curves exhibit the data at forward and backward scans, respectively. (C) The plot of mobilities as a function of V_G. The mobility values calculated from the average at the high V_G region that are shown in the orange area. (D) Typical output characteristics for *p*Tol-BTBT-C_n single crystals.

Table S6. Device parameters in parylene-based single-crystal OFET device.

Chain length *n*	Average mobility[a)] (cm^2/Vs)	Average SS (mV/dec)	Thickness of OSC layers (number of monomolecular layer) / devices
9	7.2	244	20 nm (7) / 1 TFT<br>33 nm (12) / 6 TFTs
10	6.3	99	15 nm (5) / 1 TFT, 23 nm (8) / 9 TFTs<br>28 nm (10) / 1 TFT, 32 nm (11) / 4 TFTs
11	8.6	291	18 nm (6) / 9 TFTs<br>23 nm (8) / 3 TFTs
12	8.0	106	40 nm (13) / 13 TFTs
13	10.2	357	14 nm (4) / 5 TFTs, 17 nm (5) / 1TFT<br>26nm (8) / 3 TFTs
14	6.7	158	34 nm (10) / 6 TFTs<br>38 nm (12) / 3 TFTs

a) $V_D = -30$V for n = 9, 10, 11, and 13, and $V_D = -40$V for n = 12 and 14.

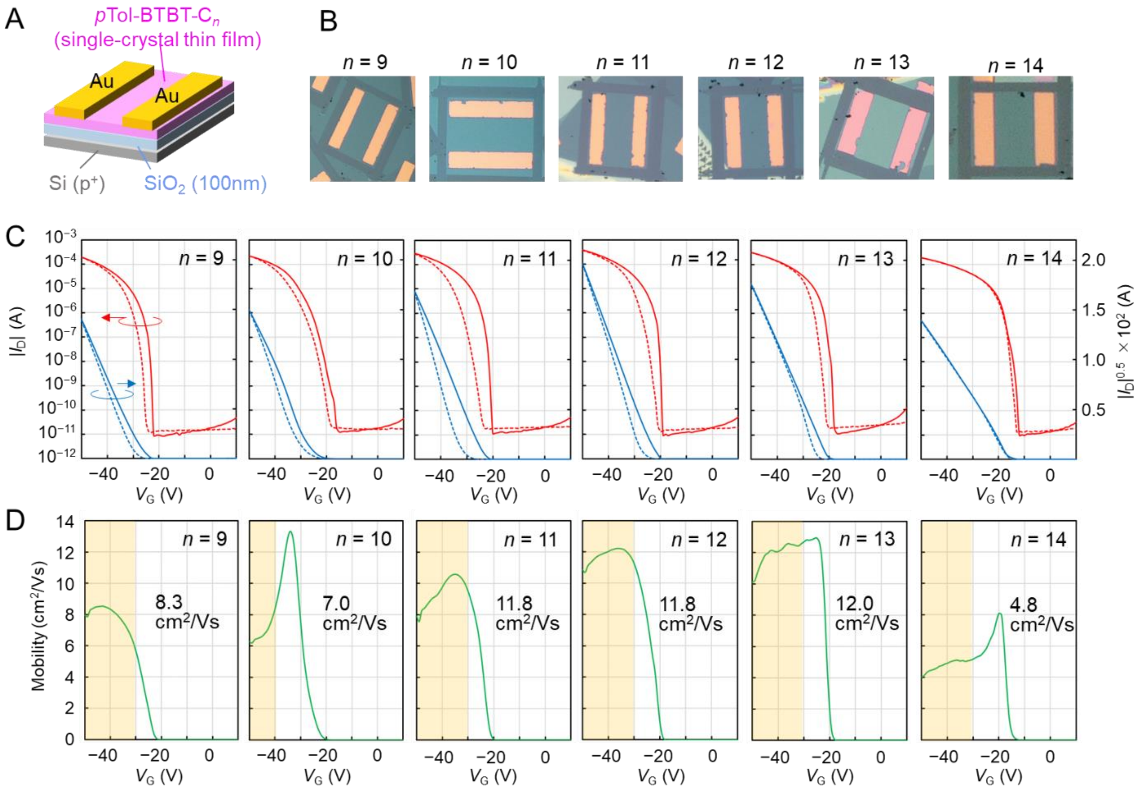

Figure S19. Sigle-crystal OFET device performance employing SiO_2 gate insulator. (A) Schematcis of the bottom-gate/top-contact (BGTC) OFET. (B) Optical microscope images of OFET devices for *p*Tol-BTBT-C*n*. (C) Typical transfer characteristics for *p*Tol-BTBT-C*n* single crystals measured at $V_D = -50V$. Solid and dashed curves exhibit the data at forward and backward scans, respectively. (D) The plot of mobilities as a function of V_G. The mobility values calculated from the average at the high V_G region that are shown in the orange area.

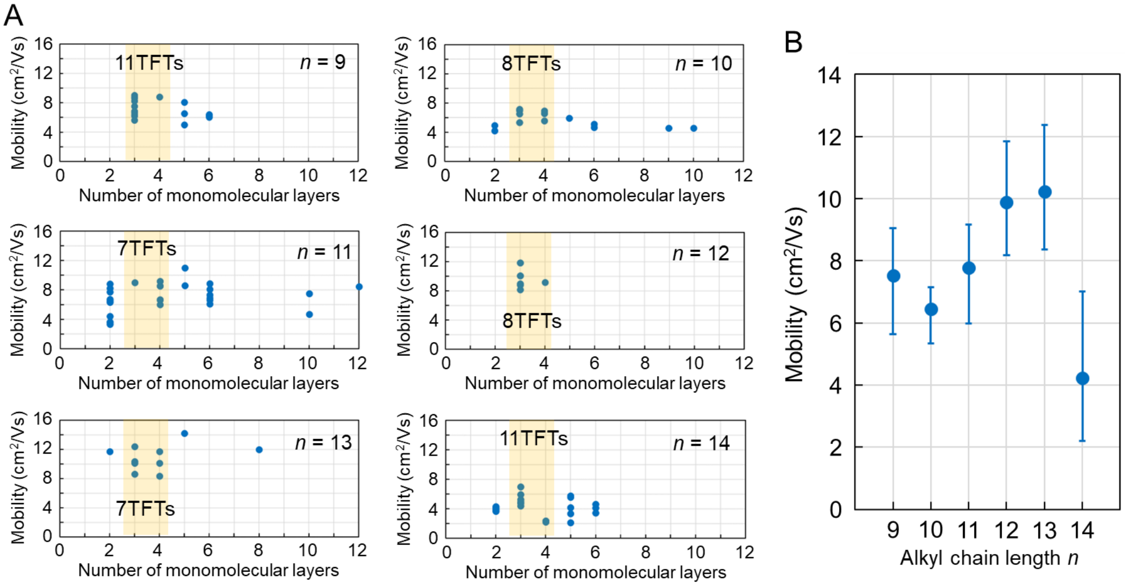

Figure S20. Film-thickness and alkyl chain length dependence on single-crystal OFET device performance employing SiO_2 gate insulator. (A) Values of the estimated BGTC device mobilities are plotted as a function of the layer number thickness of the films. (B) Alkyl chain length dependence of the mobility changes at the saturation region. Average values are used each orange area (limiting 3- and 4- layer thickness) shown in (A).

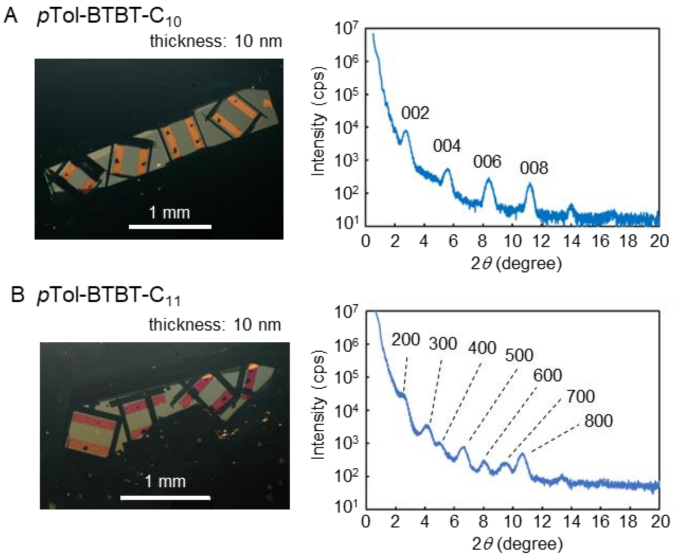

Figure S21. Optical microscope images and out-of-plane XRD profiles of the single-crystal OFET device employing SiO_2 gate insulator. (A) *p*Tol-BTBT-C_{10}. (B) *p*Tol-BTBT-C_{11}.

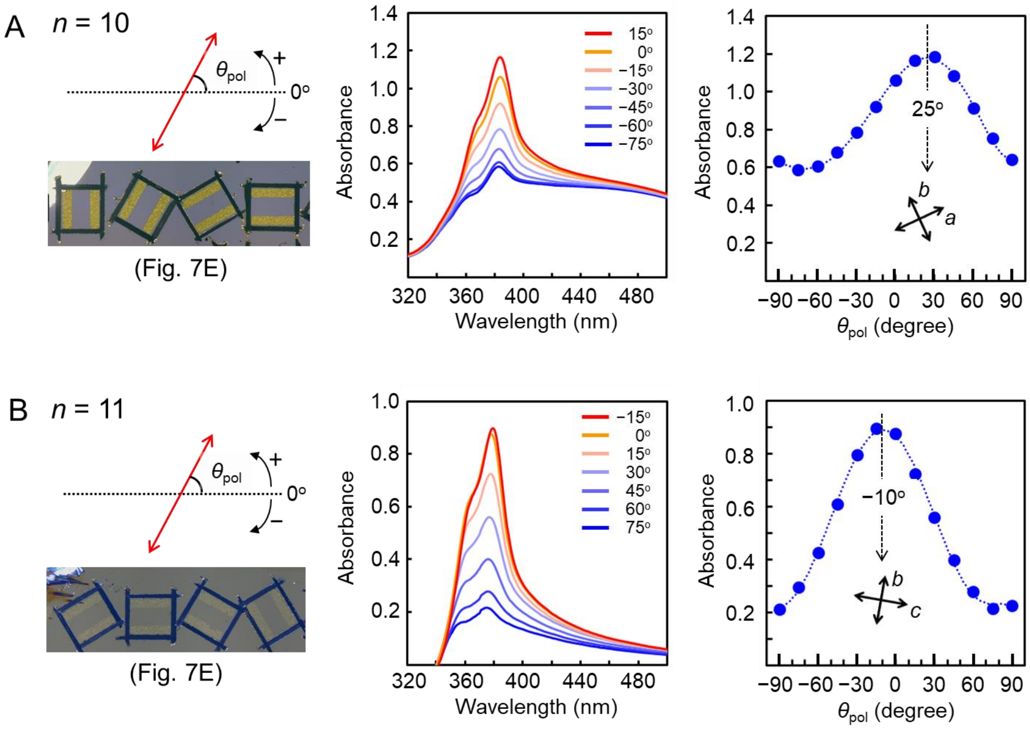

Figure S22. Determination of the crystallographic orientation in parylene-based single-crystal OFET devices. (A) *p*Tol-BTBT-C_{10}. (B) *p*Tol-BTBT-C_{11}. The definition of the polarization angle (θ_{pol}) and the polarization angle dependence of the absorbance shows left and right, respectively. The angle at which absorption becomes maximum aligns with the *a*- (or *c*-) axis direction in the thin film. The *a*- (or *c*-) axis of the single-crystal thin-film is oriented at the angle where absorption shows its maximum value.
